# Supplementary material for: Evidence of episodic positive selection in Corynebacterium diphtheriae complex of species and its implementations in identification of drug and vaccine targets
Source: PeerJ. 2022 Feb 16;10:e12662. doi: 10.7717/peerj.12662 (PMC8857904; doi:10.7717/peerj.12662)
Supplement: Supplemental Information 1 [file peerj-10-12662-s001.pdf]

TYPE (STRAIN) GENOME SERVER

JOB SUMMARY

PRINT DATE:	2021-02-09 14:54:56 +0100
JOB ID:	94fb8114-e96d-4d80-ab20-2ff779dc2a37
RESULT PAGE:	https://tygs.dsmz.de/user_results/show?guid=94fb8114-e96d-4d80-ab20-2ff779dc2a37

Table 1: Phylogenies

Publication-ready versions of both the genome-scale GBDP tree and the 16S rRNA gene sequence tree can be customized and exported either in SVG (vector graphic) or PNG format from within the phylogeny viewers in your TYGS result page. For publications the **SVG format is recommended** because it is lossless, always keeps its high resolution and can also be easily converted to other popular formats such as PDF or EPS. Please follow the link provided above!

Table 2: Identification

The below list contains the result of the TYGS species identification routine.

Explanation of remarks that might occur in the below table:

remark [R1]: The TYGS type strain database is automatically updated on an almost daily basis. However, if a particular type strain genome is not available in the TYGS database, this can have several reasons which are detailed in the FAQ. You can request an extended 16S rRNA gene analysis via the 16S tree viewer found in your result page to detect **not yet genome-sequenced** type strains relevant for your study.

remark [R2]: > 70% dDDH value (formula d_4) and (almost) minimal dDDH values for gene-content formulae d_0 and d_6 indicate a potentially unreliable identification result and should thus be checked via the 16S rRNA gene sequence similarity. Such strong deviations can, in principle, be caused by sequence contamination.

remark [R3]: G+C content difference of > 1 % indicates a potentially unreliable identification result because within species G+C content varies no more than 1 %, if computed from genome sequences (PMID: 24505073).

Strain	Conclusion	Identification result	Remark
**'Cfalsenii_DSM44353'**	belongs to known species	*Corynebacterium falsenii*	
**'Cdoosanense_DSM45436'**	belongs to known species	*Corynebacterium doosanense*	
**'Ccasei_LMGS19264'**	belongs to known species	*Brevibacterium linens*	see [R2]<br>see [R3]
**'Cbelfantii_FRC0043'**	belongs to known species	*Corynebacterium belfantii*	
**'Ccallunae_DSM20147'**	belongs to known species	*Corynebacterium callunae*	
**'Caurimucosum_ATCC700975'**	belongs to known species	*Corynebacterium nigricans*	
**'Cefficiens_YS314'**	belongs to known species	*Corynebacterium efficiens*	
**'Ckroppenstedtii_DSM44385'**	belongs to known species	*Corynebacterium kroppenstedtii*	
**'Cjeikeium_K41'**	belongs to known species	*Corynebacterium jeikeium*	
**'Ccamporealensis_DSM44610'**	belongs to known species	*Corynebacterium camporealensis*	
**'Catypicum_R2070'**	belongs to known species	*Corynebacterium atypicum*	
**'Chumireducens_DSM45392'**	belongs to known species	*Corynebacterium humireducens*	
**'Cimitans_DSM44264'**	belongs to known species	*Corynebacterium imitans*	
**'Cdeserti_GIMN1010'**	belongs to known species	*Corynebacterium deserti*	

Strain	Conclusion	Identification result	Remark
**'Cglycinophilum_AJ3170'**	belongs to known species	*Corynebacterium glyciniphilum*	
**'Cepidermidicanis_DSM45586'**	belongs to known species	*Corynebacterium epidermidicanis*	
**'Chalotolerans_YIM70093'**	belongs to known species	*Corynebacterium halotolerans*	
**'Cglutamicum_ATCC13032'**	belongs to known species	*Corynebacterium glutamicum*	
**'Cdiphtheriae_NCTC11397'**	belongs to known species	*Corynebacterium diphtheriae*	
**'Cargentoratense_DSM44202'**	belongs to known species	*Corynebacterium argentoratense*	

Table 3: Pairwise comparisons of user genomes vs. type-strain genomes

The overall number of pairwise comparisons was too large for a proper display and was thus reduced to only those comparisons having a digital DDH value ≥ 65 % in at least one of the three formulae d_0, d_4 and d_6.

The following table contains the pairwise dDDH values between your user genomes and the selected type-strain genomes. The dDDH values are provided along with their confidence intervals (C.I.) for the three different GBDP formulas:

- formula d_0 (a.k.a. GGDC formula 1): length of all HSPs divided by total genome length
- formula d_4 (a.k.a. GGDC formula 2): sum of all identities found in HSPs divided by overall HSP length
- formula d_6 (a.k.a. GGDC formula 3): sum of all identities found in HSPs divided by total genome length

Note: Formula d_4 is independent of genome length and is thus robust against the use of incomplete draft genomes. For other reasons for preferring formula d_4, see the FAQ.

Query	Subject	d_0	C.I. d_0	d_4	C.I. d_4	d_6	C.I. d_6	Diff. G+C Percent
**'Caurimucosum_ATCC700975.fna'**	*Corynebacterium nigricans* ATCC 700975	100.0	[100.0 - 100.0]	100.0	[100.0 - 100.0]	100.0	[100.0 - 100.0]	0.0
**'Cimitans_DSM44264.fna'**	*Corynebacterium imitans* NCTC 13015	100.0	[100.0 - 100.0]	100.0	[99.9 - 100.0]	100.0	[100.0 - 100.0]	0.0
**'Cepidermidicanis_DSM45586.fna'**	*Corynebacterium epidermidicanis* DSM 45586	100.0	[100.0 - 100.0]	100.0	[100.0 - 100.0]	100.0	[100.0 - 100.0]	0.0
**'Chalotolerans_YIM70093.fna'**	*Corynebacterium halotolerans* DSM 44683	100.0	[99.9 - 100.0]	100.0	[100.0 - 100.0]	100.0	[100.0 - 100.0]	0.06
**'Ckroppenstedtii_DSM44385.fna'**	*Corynebacterium kroppenstedtii* DSM 44385	100.0	[100.0 - 100.0]	100.0	[100.0 - 100.0]	100.0	[100.0 - 100.0]	0.0
**'Cglycinophilum_AJ3170.fna'**	*Corynebacterium glyciniphilum* ATCC 21341	100.0	[100.0 - 100.0]	100.0	[100.0 - 100.0]	100.0	[100.0 - 100.0]	0.0
**'Ccasei_LMGS19264.fna'**	*Corynebacterium casei* DSM 44701	100.0	[100.0 - 100.0]	100.0	[100.0 - 100.0]	100.0	[100.0 - 100.0]	0.0
**'Chumireducens_DSM45392.fna'**	*Corynebacterium humireducens* DSM 45392	100.0	[100.0 - 100.0]	100.0	[100.0 - 100.0]	100.0	[100.0 - 100.0]	0.0
**'Catypicum_R2070.fna'**	*Corynebacterium atypicum* DSM 44849	100.0	[100.0 - 100.0]	100.0	[100.0 - 100.0]	100.0	[100.0 - 100.0]	0.0
**'Cbelfantii_FRC0043.fna'**	*Corynebacterium belfantii* FRC0043	100.0	[100.0 - 100.0]	100.0	[100.0 - 100.0]	100.0	[100.0 - 100.0]	0.01
**'Cdiphtheriae_NCTC11397.fna'**	*Corynebacterium diphtheriae* NCTC 11397	100.0	[100.0 - 100.0]	100.0	[100.0 - 100.0]	100.0	[100.0 - 100.0]	0.0
**'Cglutamicum_ATCC13032.fna'**	*Corynebacterium glutamicum* ATCC 13032	100.0	[100.0 - 100.0]	100.0	[100.0 - 100.0]	100.0	[100.0 - 100.0]	0.0
**'Cfalsenii_DSM44353.fna'**	*Corynebacterium falsenii* DSM 44353	100.0	[100.0 - 100.0]	100.0	[100.0 - 100.0]	100.0	[100.0 - 100.0]	0.0
**'Cimitans_DSM44264.fna'**	*Corynebacterium imitans* DSM 44264	100.0	[100.0 - 100.0]	100.0	[100.0 - 100.0]	100.0	[100.0 - 100.0]	0.0
**'Ccallunae_DSM20147.fna'**	*Corynebacterium callunae* DSM 20147	100.0	[100.0 - 100.0]	100.0	[99.9 - 100.0]	100.0	[100.0 - 100.0]	0.0
**'Chalotolerans_YIM70093.fna'**	*Corynebacterium halotolerans* YIM 70093	100.0	[100.0 - 100.0]	100.0	[100.0 - 100.0]	100.0	[100.0 - 100.0]	0.0
**'Cdeserti_GIMN1010.fna'**	*Corynebacterium deserti* DSM 45689	100.0	[100.0 - 100.0]	100.0	[100.0 - 100.0]	100.0	[100.0 - 100.0]	0.0
**'Cargentoratense_DSM44202.fna'**	*Corynebacterium argentoratense* DSM 44202	100.0	[100.0 - 100.0]	100.0	[100.0 - 100.0]	100.0	[100.0 - 100.0]	0.0
**'Cdoosanense_DSM45436.fna'**	*Corynebacterium doosanense* DSM 45436	99.9	[99.8 - 100.0]	100.0	[99.9 - 100.0]	100.0	[99.9 - 100.0]	0.13

Query	Subject	d_0	C.I. d_0	d_4	C.I. d_4	d_6	C.I. d_6	Diff. G+C Percent
**'Cdiphtheriae_NCTC11397.fna'**	*Corynebacterium diphtheriae* DSM 44123	99.5	[99.1 - 99.8]	100.0	[100.0 - 100.0]	99.8	[99.6 - 99.9]	0.01
**'Cefficiens_YS314.fna'**	*Corynebacterium efficiens* YS-314	100.0	[100.0 - 100.0]	100.0	[100.0 - 100.0]	100.0	[100.0 - 100.0]	0.0
**'Ccamporealensis_DSM44610.fna'**	*Corynebacterium camporealensis* DSM 44610	100.0	[100.0 - 100.0]	100.0	[100.0 - 100.0]	100.0	[100.0 - 100.0]	0.0
**'Ccamporealensis_DSM44610.fna'**	*Corynebacterium camporealensis* CIP 105508	100.0	[100.0 - 100.0]	99.8	[99.7 - 99.9]	100.0	[100.0 - 100.0]	0.01
**'Chumireducens_DSM45392.fna'**	*Corynebacterium humireducens* NBRC 106098	100.0	[100.0 - 100.0]	99.8	[99.7 - 99.9]	100.0	[100.0 - 100.0]	0.22
**'Cbelfantii_FRC0043.fna'**	*Corynebacterium diphtheriae* subsp. *lausannense* CHUV2995	92.5	[89.7 - 94.6]	96.2	[94.8 - 97.2]	95.2	[93.3 - 96.6]	0.32
**'Ccasei_LMGS19264.fna'**	*Brevibacterium linens* ATCC 9172	13.1	[10.4 - 16.4]	77.1	[74.1 - 79.9]	13.6	[11.2 - 16.4]	9.08
**'Cjeikeium_K41.fna'**	*Corynebacterium jeikeium* NCTC 11913	91.7	[88.7 - 94.0]	70.6	[67.6 - 73.4]	90.8	[88.2 - 92.9]	0.08
**'Cjeikeium_K41.fna'**	*Corynebacterium jeikeium* ATCC 43734	88.7	[85.3 - 91.4]	70.2	[67.2 - 73.0]	88.2	[85.3 - 90.7]	0.28
**'Cdiphtheriae_NCTC11397.fna'**	*Corynebacterium diphtheriae* subsp. *lausannense* CHUV2995	71.1	[67.2 - 74.8]	62.9	[60.0 - 65.7]	71.8	[68.3 - 75.0]	0.42
**'Cdiphtheriae_NCTC11397.fna'**	*Corynebacterium belfantii* FRC0043	77.0	[73.1 - 80.6]	61.9	[59.0 - 64.7]	76.6	[73.2 - 79.8]	0.1
**'Cbelfantii_FRC0043.fna'**	**'Cdiphtheriae_NCTC11397.fna'**	77.0	[73.1 - 80.6]	61.9	[59.0 - 64.7]	76.6	[73.2 - 79.8]	0.1
**'Cbelfantii_FRC0043.fna'**	*Corynebacterium diphtheriae* NCTC 11397	77.0	[73.1 - 80.6]	61.9	[59.0 - 64.7]	76.6	[73.2 - 79.8]	0.1
**'Cbelfantii_FRC0043.fna'**	*Corynebacterium diphtheriae* DSM 44123	78.9	[74.9 - 82.4]	61.8	[58.9 - 64.6]	78.2	[74.8 - 81.3]	0.09
**'Caurimucosum_ATCC700975.fna'**	*Corynebacterium aurimucosum* strain DSM 44532	84.5	[80.7 - 87.6]	50.4	[47.8 - 53.0]	79.4	[76.0 - 82.4]	0.26
**'Cdiphtheriae_NCTC11397.fna'**	*Corynebacterium rouxii* FRC0190 T	75.7	[71.7 - 79.3]	49.3	[46.7 - 51.9]	71.8	[68.4 - 75.1]	0.3
**'Cbelfantii_FRC0043.fna'**	*Corynebacterium rouxii* FRC0190 T	73.0	[69.0 - 76.6]	45.4	[42.8 - 47.9]	68.2	[64.8 - 71.4]	0.39
**'Chumireducens_DSM45392.fna'**	*Corynebacterium pollutisoli* VDS	66.3	[62.5 - 69.9]	29.4	[27.0 - 31.9]	55.2	[52.1 - 58.3]	0.06
**'Cglutamicum_ATCC13032.fna'**	*Corynebacterium suranareeae* N24T	65.9	[62.1 - 69.5]	27.5	[25.1 - 30.0]	53.6	[50.5 - 56.7]	2.02

Table 4: Strains in your dataset

Joint dataset of automatically determined closest type strains (if this mode was chosen), manually selected type strains (if selected accordingly) and the provided user strains, if provided (marked in yellow).

Strain	Authority	Other deposits	Synonyms	Base pairs	Percent G+C	No. proteins	Goldstamp	Bioproject accession	Biosample accession	Assembly accession	IMG OID
Corynebacterium vitaeruminis DSM 20294	(Bechdel et al. 1928) Lanéelle et al. 1980	CCUG 28792; JCM 1323; ATCC 10234; IFO 12143; NBRC 12143; VKM B-1211; CIP 82.07; NCIB 9291; NCIMB 9291	*Brevibacterium vitaeruminis*; *Corynebacterium vityaeruminis*; *Flavobacterium vitarumen*	2931 780	65.5	2577	Gp0023683	PRJNA172966	SAMN03081455	GCA_000550805	2558860221
Corynebacterium falsenii DSM 44353	Sjödén et al. 1998 emend. Nouioui et al. 2018	CCUG 33651; JCM 11949; CIP 105466; Y13024	*Corynebacterium falsenii*	2719 559	63.2	2306	Gp0086746	PRJNA235944	SAMN02641485	GCA_000525655	2571042744
Corynebacterium lubricantis DSM 45231	Kämpfer et al. 2009 emend. Nouioui et al. 2018	CCUG 56567; JCM 16607; CCM 7546; KSS-3Se	*Corynebacterium lubricantis*	2945 292	58.6	2818	Gp0013695	PRJNA165249	SAMN02256424	GCA_000379425	2515154018
Corynebacterium doosanense DSM 45436	Lee et al. 2009 emend. Nouioui et al. 2018	KCTC 19568; CCUG 57284; CAU 212	*Corynebacterium doosanense*	2649 019	66.9	2590	Gp0013691	PRJNA165377	SAMN02256506	GCA_000372245	2515154029
Brevibacterium linens ATCC 9172	(Wolff 1910) Breed 1953	NRRL B-4210; DSM 20425; JCM 1327; IFO 12142; NBRC 12142; VKM Ac-2112; CIP 101125; HAMBI 2038	*Bacterium linens*; *Brevibacterium linens*	3959 351	64.8	3518		PRJEB19834	SAMEA103891064	GCA_900169165	

Strain	Authority	Other deposits	Synonyms	Base pairs	Percent G+C	No. proteins	Goldstamp	Bioproject accession	Biosample accession	Assembly accession	IMG OID
Corynebacterium mastitidis DSM 44356	Fernandez-Garayzabal et al. 1997 emend. Nouioui et al. 2018	LMG 19040; CCUG 38654; CECT 4843; JCM 12269; IFO 16160; NBRC 16160; CIP 105509; strain S-8	*Corynebacterium mastitidis*	2371 714	69.0	2241	Gp0013697	PRJNA169809	SAMN02441393	GCA_000375365	2515154131
Corynebacterium pilosum DSM 20521	Yanagawa and Honda 1978	CCUG 27193; DSM 20521; JCM 3714; ATCC 29592; IFO 15285; NBRC 15285; NCTC 11862; CIP 103422	*Corynebacterium pilosum*	2532 067	60.7	2387	Gp0013698	PRJNA169769	SAMN02441706	GCA_000373805	2515154153
Corynebacterium fournieri Marseille-P2948	Diop et al. 2018	DSM 103271; CSUR P2948	*Corynebacterium fournieri*	2357 034	65.0	2305	Gp0370410	PRJEB20393	SAMEA103975581	GCA_900176865	
Corynebacterium riegelii DSM 44326	Funke et al. 1998	CCUG 38180; JCM 10389; ATCC 700782; CIP 105310; DMMZ 2415	*Corynebacterium riegelii*	2519 232	60.4	2283		PRJNA231221	SAMN16357283		
Corynebacterium anserum 23H37-10	Liu et al. 2021	GDMCC 1.1737; KACC 21672	*Corynebacterium anserum*	2208 656	55.2	1764		PRJNA595090	SAMN13546099	GCA_014262665	
Corynebacterium godavarianum LMG 29598	Jani et al. 2018	MCC 3388; KCTC 39803; PRD07	*Corynebacterium godavarianum*	2521 298	65.6	2235		PRJNA555895	SAMN12335367	GCA_007559235	
Corynebacterium endometrii LMM-1653T	Ballas et al. 2020	LMG-31164; CCM 8952	*Corynebacterium endometrii*	2477 061	60.9	2189		PRJNA224116	SAMN11357123	GCF_004795735	

Strain	Authority	Other deposits	Synonyms	Base pairs	Percent G+C	No. proteins	Goldstamp	Bioproject accession	Biosample accession	Assembly accession	IMG OID
Corynebacterium aurimucosum strain DSM 44532	Yassin et al. 2002 emend. Daneshvar et al. 2004	NRRL B-24143; CCUG 47449; JCM 11766; IMMIB D-1488	*Corynebacterium aurimucosum*	2737 787	60.3	2417		PRJNA231221	SAMN16357278	GCA_000626615	
Corynebacterium alimapuense CCUG 69366	Claverias et al. 2019	NCIMB 15118; VA37-3	*Corynebacterium alimapuense*	2281 535	57.1	2040	Gp0385727	PRJNA305687	SAMN08535650	GCA_003716585	
Corynebacterium phoceense MC1	Cresci et al. 2016	DSM 100570; CSUR P1905	*Corynebacterium phoceense*	2772 735	63.2	2701		PRJNA224116	SAMEA4059842	GCF_900092335	
Corynebacterium diphtheriae subsp. *lausannense* CHUV2995	Tagini et al. 2019	CCUG 72509; DSM 107520	*Corynebacterium diphtheriae* subsp. *lausannense*	3060 363	53.9	3145	Gp0442955	PRJEB24256	SAMEA104679569	GCA_900312965	
Corynebacterium belfantii FRC0043	Dazas et al. 2018	DSM 105776; CIP 111412	*Corynebacterium belfantii*	2598 827	53.6	2557	Gp0364753	PRJEB22103	SAMEA104208677	GCA_900205605	
Corynebacterium callunae DSM 20147	(Lee and Good 1963) Yamada and Komagata 1972 emend. Nouioui et al. 2018	CCUG 28793; JCM 9489; ATCC 15991; IFO 15359; NBRC 15359; CIP 104277; HAMBI 2053; NCFB 10338; NCIB 10338; NCIMB 10338	*Corynebacterium callunae*	2890 884	52.4	2679	Gp0013686	PRJNA185570	SAMN02441249	GCA_000420585	2522572159
Corynebacterium urogenitale DSM 108747	Ballas et al. 2020	LMG 31163; LMM-1652	*Corynebacterium urogenitale*	2351 892	59.9	2058		PRJNA224116	SAMN12924940	GCF_009026825	
Corynebacterium choanae CCM 8831	Busse et al. 2019	200CH; LMG 30628; CCUG 72166	*Corynebacterium choanae*	2986 773	57.0	2308	Gp0443147	PRJNA432431	SAMN08448940	GCA_003813965	

Strain	Authority	Other deposits	Synonyms	Base pairs	Percent G+C	No. proteins	Goldstamp	Bioproject accession	Biosample accession	Assembly accession	IMG OID
Corynebacterium frankenforstense ST18	Wiertz et al. 2013 emend. Nouioui et al. 2018	CCUG 63371; DSM 45800	*Corynebacterium frankenforstense*	2604 152	71.5	1801	Gp0118685	PRJNA232093	SAMN02991553	GCA_001941485	
Corynebacterium flavescens DSM 20296	Barksdale et al. 1979 emend. Nouioui et al. 2018	8 of Orla-Jensen; LMG 4046; CCUG 28791; DSM 20296; JCM 1317; ATCC 10340; IFO 14136; NBRC 14136; VKM Ac-1956; CIP 69.5; NCCB 42012; NCDO 1320; NCFB 1320; NCIB 8707; NCIMB 8707	*Corynebacterium flavescens*	2758 653	59.9	2202	Gp0118684	PRJNA242338	SAMN02996497	GCA_001941465	
Corynebacterium sphenisci DSM 44792	Goyache et al. 2003 emend. Nouioui et al. 2018	CCUG 46398; CECT 5990; JCM 12270	*Corynebacterium sphenisci*	2594 799	74.7	1827	Gp0118687	PRJNA232092	SAMN02996499	GCA_001941505	
Corynebacterium aquilae S-613	Fernández-Garayzábal et al. 2003 emend. Nouioui et al. 2018	CCUG 46511; CECT 5993; DSM 44791; JCM 12268	*Corynebacterium aquilae*	2926 436	60.9	2013	Gp0118683	PRJNA230273	SAMN02996496	GCA_001941445	
Corynebacterium stationis DSM 20302	(ZoBell and Upham 1944) Bernard et al. 2010	CCUG 43497; JCM 11611; ATCC 14403; IFO 12144; NBRC 12144; VKM B-1228; CIP 104228	*Achromobacter stationis*; *Brevibacterium stationis*; *Corynebacterium stationis*	2862 233	54.9	2380	Gp0118688	PRJNA242207	SAMN02996501	GCA_001941345	

Strain	Authority	Other deposits	Synonyms	Base pairs	Percent G+C	No. proteins	Goldstamp	Bioproject accession	Biosample accession	Assembly accession	IMG OID
Corynebacterium appendicis DSM 44531	Yassin et al. 2002 emend. Nouioui et al. 2018	NRRL B-24151; CCUG 48298; JCM 11765; IMMIB R-3491	*Corynebacterium appendicis*	2248 056	64.3	2155	Gp0131803	PRJEB18828	SAMN05444817	GCA_900156665	2681813517
Corynebacterium afermentans DSM 44280	Riegel et al. 1993 emend. Nouioui et al. 2018	CCUG 32103; JCM 10390; ATCC 51403; CIP 103499; LCDC 88199	*Corynebacterium afermentans*; *Corynebacterium afermentans* subsp. *afermentans*	2326 687	64.9	2171	Gp0131801	PRJEB18848	SAMN05421802	GCA_900156035	2681813557
Corynebacterium mooreparkense DSM 44702	Brennan et al. 2001 emend. Nouioui et al. 2018	LMG S-19265; JCM 12073; CIP 107183; DPC 5310; NCIMB 30131	*Corynebacterium mooreparkense*	3433 007	67.1	3039	Gp0008259	PRJNA50001	SAMN02603088	GCA_000179395	2511231114
Corynebacterium nigricans ATCC 700975	Shukla et al. 2004 emend. Nouioui et al. 2018	CCUG 48176; DSM 44827; JCM 12684; CIP 107346; CN-1	*Corynebacterium nigricans*	2819 226	60.6	2551	Gp0004919	PRJNA37279	SAMN02603064	GCA_000022905	643692018
Corynebacterium tuberculostearicum DSM 44922	Feurer et al. 2004	CCUG 45418; JCM 13389; ATCC 35692; CIP 107291; LDC-20; Medalle X	*Corynebacterium tuberculostearicum*	2453 172	59.7	2326	Gp0126956	PRJNA347115	SAMN05878002	GCA_013408445	2833975288

Strain	Authority	Other deposits	Synonyms	Base pairs	Percent G+C	No. proteins	Goldstamp	Bioproject accession	Biosample accession	Assembly accession	IMG OID
Corynebacterium efficiens YS-314	Fudou et al. 2002 emend. Nouioui et al. 2018	AJ 12310; CCUG 47130; CCUG 48037; DSM 44549; JCM 11189; NBRC 100395	*Corynebacterium efficiens*	3219505	63.0	2998	Gp0000636	PRJNA305	SAMD00061103	GCA_000011305	644736345
Corynebacterium kroppenstedtii DSM 44385	Collins et al. 1998 emend. Nouioui et al. 2018	CCUG 35717; JCM 11950; CIP 105744	*Corynebacterium kroppenstedtii*	2446804	57.5	2018	Gp0000013	PRJNA38011	SAMN02603033	GCA_000023145	643692019
Corynebacterium urinapleomorphum Marseille-P2799	Niang et al. 2019	DSM103272; CSURP2799	*Corynebacterium urinapleomorphum*	2259535	63.4	2097		PRJEB18932	SAMEA47264668	GCA_900155535	
Corynebacterium maris DSM 45190	Ben-Dov et al. 2009	LMG 24561; JCM 17018; Coryn-1	*Corynebacterium maris*	2833547	66.6	2584	Gp0023680	PRJNA172964	SAMN02603057	GCA_000442645	2561511185
Corynebacterium silvaticum KL0182	Dangel et al. 2020	LMG 31313; DSM 109166; CIP 111672	*Corynebacterium silvaticum*	2548487	54.4	2017		PRJNA517029	SAMN10039578	GCA_004382825	
Corynebacterium resistens DSM 45100	Otsuka et al. 2005 emend. Nouioui et al. 2018	CCUG 50093; JCM 12819; GTC 2026; SICGH 158	*Corynebacterium resistens*	2601311	57.1	2171	Gp0003835	PRJNA39683	SAMN02603065	GCA_000177535	650716029
Corynebacterium senegalense Marseille-P4329	Ndiaye et al. 2019	CSURP4329	*Corynebacterium senegalense*	2310902	68.7	2173	Gp0359219	PRJEB24601	SAMEA4664250	GCA_900411315	

Strain	Authority	Other deposits	Synonyms	Base pairs	Percent G+C	No. proteins	Goldstamp	Bioproject accession	Biosample accession	Assembly accession	IMG OID
Corynebacterium striatum NBRC 15291	(Chester 1901) Eberson 1918 emend. Nouioui et al. 2018	CCUG 27949; DSM 20668; JCM 9390; ATCC 6940; IFO 15291; NBRC 15291; NCTC 764; CIP 81.15	*Bacterium striatum*; *Corynebacterium striatum*	3106 742	59.1	2949		PRJDB8042	SAMD00169825	GCA_006538485	
Corynebacterium flavescens NBRC 14136	Barksdale et al. 1979 emend. Nouioui et al. 2018	8 of Orla-Jensen; LMG 4046; CCUG 28791; DSM 20296; JCM 1317; ATCC 10340; IFO 14136; NBRC 14136; VKM Ac-1956; CIP 69.5; NCCB 42012; NCDO 1320; NCFB 1320; NCIB 8707; NCIMB 8707	*Corynebacterium flavescens*	2633 833	60.0	2430		PRJDB6000	SAMD00097245	GCA_006539465	
Corynebacterium xerosis ATCC 373	(Lehmann and Neumann 1896) Lehmann and Neumann 1899 emend. Nouioui et al. 2018	CCUG 27544; DSM 20743; JCM 1971; ATCC 373; IFO 16721; NBRC 16721; NCTC 11861; CIP 100653	*Bacillus xerosis*; *Corynebacterium xerosis*	2661 590	69.5	1843		PRJNA280206	SAMN03458390	GCA_000988235	

Strain	Authority	Other deposits	Synonyms	Base pairs	Percent G+C	No. proteins	Goldstamp	Bioproject accession	Biosample accession	Assembly accession	IMG OID
Corynebacterium diphtheriae DSM 44123	(Kruse 1886) Lehmann and Neumann 1896 emend. Nouioui et al. 2018	DSM 44123; ATCC 27010; NCTC 11397; CIP 100721	*Bacillus diphtheriae*; *Corynebacterium diphtheriae*; *Corynebacterium diphtheriae* subsp. *diphtheriae*	2364 574	53.5	2116		PRJNA296455	SAMN04099181	GCA_001913265	
Corynebacterium minutissimum NCTC10288	(ex Sarkany et al. 1962) Collins and Jones 1983 emend. Nouioui et al. 2018	CCUG 541; DSM 20651; JCM 9387; ATCC 23348; IFO 15361; NBRC 15361; NCTC 10288; CIP 100652	*Corynebacterium minutissimum*	2695 970	59.9	2463		PRJEB6403	SAMEA4030732	GCA_900478045	
Corynebacterium renale NCTC7448	(Migula 1900) Ernst 1906 emend. Nouioui et al. 2018	CCUG 27542; DSM 20688; JCM 9391; ATCC 19412; IFO 15290; NBRC 15290; NCTC 7448; CIP 103421; HAMBI 2321	*Bacterium renale*; *Corynebacterium renale*	2335 089	59.1	2161		PRJEB6403	SAMEA4030736	GCA_900478035	
Corynebacterium imitans NCTC 13015	Funke et al. 1997 emend. Nouioui et al. 2018	2023; CCUG 36877; DSM 44264; JCM 10386; ATCC 700354; IFO 16163; NBRC 16163; NBRC 100416; NCTC 13015; CIP 105130	*Corynebacterium imitans*	2565 606	64.3	2347		PRJEB6403	SAMEA4535761	GCA_900187215	

Strain	Authority	Other deposits	Synonyms	Base pairs	Percent G+C	No. proteins	Goldstamp	Bioproject accession	Biosample accession	Assembly accession	IMG OID
Corynebacterium urealyticum NCTC12011	Pitcher et al. 1992 emend. Nouioui et al. 2018	LMG 19041; CCUG 18158; DSM 7109; JCM 10395; ATCC 43042; NCTC 12011; CIP 103524	*Corynebacterium urealyticum*	2377 532	64.2	2005		PRJEB6403	SAMEA4530651	GCA_900187235	
Corynebacterium jeikeium NCTC 11913	Jackman et al. 1988 emend. Nouioui et al. 2018	CCUG 27192; DSM 46361; DSM 7171; JCM 9384; ATCC 43734; NCTC 11913; CIP 103337	*Corynebacterium jeikeium*	2526 027	61.4	2220		PRJEB6403	SAMEA4017703	GCA_900461185	
Corynebacterium minutissimum NCTC 10289	(ex Sarkany et al. 1962) Collins and Jones 1983 emend. Nouioui et al. 2018	CCUG 541; DSM 20651; JCM 9387; ATCC 23348; IFO 15361; NBRC 15361; NCTC 10288; CIP 100652	*Corynebacterium minutissimum*	2736 326	59.9	2571		PRJEB6403	SAMEA104016176	GCA_900447475	
Corynebacterium pilosum NCTC 11862	Yanagawa and Honda 1978	CCUG 27193; DSM 20521; JCM 3714; ATCC 29592; IFO 15285; NBRC 15285; NCTC 11862; CIP 103422	*Corynebacterium pilosum*	2593 653	60.7	2398		PRJEB6403	SAMEA4530650	GCA_900447205	

Strain	Authority	Other deposits	Synonyms	Base pairs	Percent G+C	No. proteins	Goldstamp	Bioproject accession	Biosample accession	Assembly accession	IMG OID
Corynebacterium striatum NCTC 764	(Chester 1901) Eberson 1918 emend. Nouioui et al. 2018	CCUG 27949; DSM 20668; JCM 9390; ATCC 6940; IFO 15291; NBRC 15291; NCTC 764; CIP 81.15	*Bacterium striatum*; *Corynebacterium striatum*	2924 414	59.1	2730		PRJEB6403	SAMEA4521472	GCA_900447675	
Corynebacterium spheniscorum CCUG 45512	Goyache et al. 2003 emend. Nouioui et al. 2018	CCUG 45512; CECT 5986; DSM 44757; JCM 12271; PG 39	*Corynebacterium spheniscorum*	2456 378	57.5	2059		PRJNA563568	SAMN12771121	GCA_008693095	
Corynebacterium flavescens CCUG 28791	Barksdale et al. 1979 emend. Nouioui et al. 2018	8 of Orla-Jensen; LMG 4046; CCUG 28791; DSM 20296; JCM 1317; ATCC 10340; IFO 14136; NBRC 14136; VKM Ac-1956; CIP 69.5; NCCB 42012; NCDO 1320; NCFB 1320; NCIB 8707; NCIMB 8707	*Corynebacterium flavescens*	2687 049	60.0	2369		PRJNA563568	SAMN12771119	GCA_008693105	
Corynebacterium humireducens NBRC 106098	Wu et al. 2011 emend. Nouioui et al. 2018	DSM 45392; NBRC 106098; CGMCC 2452; MFC-5	*Corynebacterium humireducens*	2609 893	68.8	2525	Gp0023682	PRJDB440	SAMD00046477	GCA_001571025	

Strain	Authority	Other deposits	Synonyms	Base pairs	Percent G+C	No. proteins	Goldstamp	Bioproject accession	Biosample accession	Assembly accession	IMG OID
Corynebacterium camporealensis CIP 105508	Fernández-Garayzábal et al. 1998 emend. Nouioui et al. 2018	CCUG 39412; CECT 4897; DSM 44610; JCM 11664; ATCC BAA-77; CIP 105508; strain CRS-51	*Corynebacterium camporealensis*	2440 812	59.4	1684	Gp0149906	PRJNA262863	SAMN03092874	GCA_000766885	
Corynebacterium heidelbergense DSM 104638T	Braun et al. 2018		*Corynebacterium heidelbergense*	2287 330	65.1	2027	Gp0393075	PRJNA419723	SAMN08095970	GCA_003285565	
Corynebacterium tuscaniense CCUG 51321	Riegel et al. 2006	DSM 45101; JCM 15294; ATCC BAA-1141; ISS-5309	*Corynebacterium tuscaniense*	2232 117	59.4	2073		PRJNA224116	SAMN12771122	GCF_008693065	
Corynebacterium halotolerans DSM 44683	Chen et al. 2004 emend. Nouioui et al. 2018	DSM 44683; JCM 12676; CCTCC AA 001024; YIM 70093	*Corynebacterium halotolerans*	3202 499	68.4	2914	Gp0013694	PRJNA215338	SAMN02743911	GCA_000688435	2556921045
Corynebacterium variabile NBRC 15286	(Müller 1961) Collins 1987	NRRL B-4201; CCUG 45246; DSM 20132; JCM 2154; ATCC 15753; IFO 15286; VKM Ac-1122; CIP 102112; HAMBI 1872; NCIB 9455; NCIMB 9455	*Arthrobacter variabilis*; *Corynebacterium variabile*	3170 194	67.4	2983		PRJDB6001	SAMD00097555	GCA_006539825	
Corynebacterium tapiri LMG 28165	Baumgardt et al. 2015	2385/12; CCUG 65456	*Corynebacterium tapiri*	2246 156	62.3	2045		PRJNA545602	SAMN11928016	GCA_006334925	

Strain	Authority	Other deposits	Synonyms	Base pairs	Percent G+C	No. proteins	Goldstamp	Bioproject accession	Biosample accession	Assembly accession	IMG OID
Corynebacterium rouxii FRC0190 T	Badell et al. 2020	DSM 110354; CIP 111752	*Corynebacterium rouxii*	2451 019	53.2	2365		PRJNA224116	SAMEA5992727	GCF_902702935	
Corynebacterium mucifaciens ATCC 700355	Funke et al. 1997	CCUG 36878; DSM 44265; JCM 10384; CIP 105129; DMMZ 2278	*Corynebacterium mucifaciens*	2180 241	65.5	2028		PRJNA622446	SAMN14517857	GCA_012396315	
Corynebacterium suranareeae N24T	Nantapong et al. 2020	TBRC 5845; NBRC 113465	*Corynebacterium suranareeae*	3537 507	51.8	3194		PRJNA224116	SAMD00000552	GCF_002355155	
Corynebacterium spheniscorum DSM 44757	Goyache et al. 2003 emend. Nouioui et al. 2018	CCUG 45512; CECT 5986; DSM 44757; JCM 12271; PG 39	*Corynebacterium spheniscorum*	2451 870	57.5	2156	Gp0102878	PRJNA262359	SAMN05660282	GCA_900113445	2599185219
Corynebacterium cystitidis DSM 20524	Yanagawa and Honda 1978 emend. Nouioui et al. 2018	CCUG 28794; DSM 20524; JCM 3715; ATCC 29593; IFO 15284; NBRC 15284; NCTC 11863; CIP 103424	*Corynebacterium cystitidis*	2943 980	57.0	2789	Gp0102855	PRJNA262324	SAMN05661109	GCA_900111265	2599185266
Corynebacterium crudilactis DSM 100882	Zimmermann et al. 2016	LMG 29813; CCUG 69192; JZ16	*Corynebacterium crudilactis*	3217 286	51.7	2825	Gp0203249	PRJNA321311	SAMN04990137	GCA_001643015	
Corynebacterium hadale NBT06-6	Wei et al. 2018	MCCC 1K03347; DSM 105365	*Corynebacterium hadale*	2679 199	65.2	2362	Gp0374689	PRJNA396693	SAMN07460149	GCA_002273005	

Strain	Authority	Other deposits	Synonyms	Base pairs	Percent G+C	No. proteins	Goldstamp	Bioproject accession	Biosample accession	Assembly accession	IMG OID
Corynebacterium pollutisoli VDS	Negi et al. 2016	MCC 2722; KCTC 39687; DSM 100104; VDS11	*Corynebacterium pollutisoli*	2535 040	68.5	2430	Gp0156991	PRJEB20263	SAMN06295981	GCA_900177745	
Corynebacterium yudongzhengii 2183	Zhu et al. 2020	DSM 106264; CGMCC 1.16416	*Corynebacterium yudongzhengii*	2511 302	64.9	2142		PRJNA431327	SAMN08388720	GCA_003065405	
Corynebacterium provencense SN15	Lo et al. 2019	DSM 101074; CSURP216 1; Marseille-P2161	*Corynebacterium provencense*	3075 769	66.9	2799		PRJEB12691	SAMEA3869306	GCA_900049755	
Corynebacterium bouchesdurhone nse SN14	Lo et al. 2019	DSM 100846; CSURP206 7; Marseille-P2067	*Corynebacterium bouchesdurhone nse*	2255 535	68.0	2147		PRJEB13138	SAMEA3905754	GCA_900078305	
Corynebacterium pseudotuberculo sis ATCC 19410	(Buchanan 1911) Eberson 1918 emend. Nouioui et al. 2018	CCUG 2806; DSM 20689; JCM 9389; ATCC 19410; IFO 15363; NBRC 15363; NCTC 3450; CIP 102968	*Bacillus pseudotuberculo sis*; *Corynebacterium pseudotuberculo sis*	2337 763	52.2	2146	Gp0223239	PRJNA382169	SAMN06701041	GCA_002155265	
Dermabacter jinjuensis 32	Park et al. 2016	DSM 101003; NCCP 16133	*Dermabacter jinjuensis*	2398 786	62.9	1996	Gp0266532	PRJNA407870	SAMN07665299	GCA_002443115	
Corynebacterium jeddahense JCB	Edouard et al. 2017	DSM 45997; CSUR P778	*Corynebacterium jeddahense*	2472 125	67.2	2341	Gp0101187	PRJEB4941	SAMEA3138931	GCA_000577555	

Strain	Authority	Other deposits	Synonyms	Base pairs	Percent G+C	No. proteins	Goldstamp	Bioproject accession	Biosample accession	Assembly accession	IMG OID
Corynebacterium pseudotuberculosis DSM 20689	(Buchanan 1911) Eberson 1918 emend. Nouioui et al. 2018	CCUG 2806; DSM 20689; JCM 9389; ATCC 19410; IFO 15363; NBRC 15363; NCTC 3450; CIP 102968	*Bacillus pseudotuberculosis*; *Corynebacterium pseudotuberculosis*	2338546	52.2	2084	Gp0220522	PRJNA442833	SAMN08778220	GCA_003634885	2756170169
Corynebacterium ulcerans NCTC 7910	(ex Gilbert and Stewart 1927) Riegel et al. 1995	CCUG 2708; DSM 46325; JCM 10387; ATCC 51799; CIP 106504	*Corynebacterium ulcerans*	2453761	53.3	2178	Gp0262745	PRJEB6403	SAMEA4504038	GCA_900187135	
Corynebacterium aquatimens DSM 45632	Aravena-Román et al. 2012	CCUG 61574; IMMIB L-2475	*Corynebacterium aquatimens*	2525265	61.0	2230	Gp0305023				2880529280
Corynebacterium mycetoides DSM 20632	(ex Castellani 1942) Collins 1983 emend. Nouioui et al. 2018	CCUG 27538; JCM 9388; ATCC 43995; IFO 15289; NBRC 15289; NCTC 9864; CIP 55.51	*Corynebacterium mycetoides*	2266370	66.6	2129	Gp0116506	PRJNA303721	SAMN04488535	GCA_900103625	2634166344
Corynebacterium timonense DSM 45434	Merhej et al. 2009 emend. Nouioui et al. 2018	5401744; CCUG 53856; DSM 45434; CIP 109424; CSUR P20	*Corynebacterium timonense*	2633085	66.6	2470	Gp0116508	PRJNA303719	SAMN04488539	GCA_900105305	2636416018

Strain	Authority	Other deposits	Synonyms	Base pairs	Percent G+C	No. proteins	Goldstamp	Bioproject accession	Biosample accession	Assembly accession	IMG OID
Corynebacterium ammoniagenes DSM 20306	(Cooke and Keith 1927) Collins 1987 emend. Nouioui et al. 2018	CCUG 38796; JCM 1305; ATCC 6871; IFO 12612; NBRC 12612; VKM B-672; CIP 101283; NCCB 60030; NCIB 8143; NCIMB 8143	*Bacterium ammoniagenes*; *Brevibacterium ammoniagenes*; *Corynebacterium ammoniagenes*	2759 010	55.6	2654	Gp0003415	PRJNA38319	SAMN00189098	GCA_000164115	647000230
Corynebacterium pyruviciproducens ATCC BAA-1742	Tong et al. 2010	06-1773O; CCUG 57046; DSM 45585; WAL 19168	*Corynebacterium pyruviciproducens*	2703 797	61.2	2447	Gp0012480	PRJNA78965	SAMN02596973	GCA_000411375	2541047000
Corynebacterium accolens ATCC 49725	Neubauer et al. 1991 emend. Nouioui et al. 2018	CNCTC Th 1/57; CCUG 28779; DSM 44278; JCM 8331; CIP 104783	*Corynebacterium accolens*	2406 629	59.7	2333	Gp0003417	PRJNA31443	SAMN00002226	GCA_000159115	643886058
Corynebacterium lipophiloflavum DSM 44291	Funke et al. 1997	CCUG 37336; JCM 10383; ATCC 700352; CIP 105127; DMMZ 1944	*Corynebacterium lipophiloflavum*	2287 535	64.9	2371	Gp0003463	PRJNA31447	SAMN00001476	GCA_000159635	643886002

Strain	Authority	Other deposits	Synonyms	Base pairs	Percent G+C	No. proteins	Goldstamp	Bioproject accession	Biosample accession	Assembly accession	IMG OID
Corynebacterium striatum ATCC 6940	(Chester 1901) Eberson 1918 emend. Nouioui et al. 2018	CCUG 27949; DSM 20668; JCM 9390; ATCC 6940; IFO 15291; NBRC 15291; NCTC 764; CIP 81.15	*Bacterium striatum*; *Corynebacterium striatum*	2717 381	59.4	2677	Gp0003467	PRJNA31449	SAMN00001507	GCA_000159135	643886057
Rothia mucilaginosa ATCC 25296	(Bergan and Kocur 1982) Collins et al. 2000 emend. Nouioui et al. 2018	CCUG 20962; DSM 20746; JCM 10910; IFO 15673; NBRC 15673; NCTC 10663; CCM 2417; CIP 71.14	*Rothia mucilaginosa*; *Stomatococcus mucilaginosus*	2255 154	59.5	1737	Gp0004126	PRJNA31405	SAMN00001919	GCA_000175615	645058800
Corynebacterium amycolatum ATCC 49368	Collins et al. 1988	CCUG 35685; DSM 6922; JCM 7447; IFO 15207; NBRC 15207; CIP 103452; NCFB 2768; NCIMB 13130; S160	*Corynebacterium amycolatum*	2448 224	58.7	2128		PRJNA224116	SAMN07741515	GCA_014335175	
Corynebacterium atypicum DSM 44849	Hall et al. 2003 emend. Nouioui et al. 2018	CCUG 45804; JCM 12368; CIP 107431; R-2070	*Corynebacterium atypicum*	2359 433	65.4	1578	Gp0099220	PRJNA255205	SAMN02911287	GCA_000732945	
Corynebacterium auriscanis CIP 106629	Collins et al. 2000 emend. Nouioui et al. 2018	CCUG 39938; DSM 44609; JCM 12369; M598/96/1	*Corynebacterium auriscanis*	2568 862	58.5	1537	Gp0107708	PRJNA262562	SAMN03106126	GCA_000767255	

Strain	Authority	Other deposits	Synonyms	Base pairs	Percent G+C	No. proteins	Goldstamp	Bioproject accession	Biosample accession	Assembly accession	IMG OID
Corynebacterium camporealensis DSM 44610	Fernández-Garayzábal et al. 1998 emend. Nouioui et al. 2018	CCUG 39412; CECT 4897; DSM 44610; JCM 11664; ATCC BAA-77; CIP 105508; strain CRS-51	*Corynebacterium camporealensis*	2451 722	59.4	2249	Gp0110294	PRJNA276036	SAMN03365263	GCA_000980815	
Corynebacterium casei DSM 44701	Brennan et al. 2001 emend. Nouioui et al. 2018	LMG S-19264; JCM 12072; CIP 107182; DPC 5298; NCIMB 30130	*Corynebacterium casei*	3132 211	55.7	2809	Gp0036841	PRJNA186910	SAMN03081454	GCA_000550785	2558860241
Corynebacterium humireducens DSM 45392	Wu et al. 2011 emend. Nouioui et al. 2018	DSM 45392; NBRC 106098; CGMCC 2452; MFC-5	*Corynebacterium humireducens*	2681 312	68.6	2545	Gp0023681	PRJNA172965	SAMN03283197	GCA_000819445	
Corynebacterium imitans DSM 44264	Funke et al. 1997 emend. Nouioui et al. 2018	2023; CCUG 36877; DSM 44264; JCM 10386; ATCC 700354; IFO 16163; NBRC 16163; NBRC 100416; NCTC 13015; CIP 105130	*Corynebacterium imitans*	2565 109	64.3	2013	Gp0094474	PRJNA246650	SAMN02950575	GCA_000739455	
Corynebacterium marinum DSM 44953	Du et al. 2010 emend. Nouioui et al. 2018	7015; NRRL B-24779; DSM 44953; CGMCC 1.6998; D7015	*Corynebacterium marinum*	2729 219	67.8	2550	Gp0023707	PRJNA172963	SAMN02800399	GCA_000835165	

Strain	Authority	Other deposits	Synonyms	Base pairs	Percent G+C	No. proteins	Goldstamp	Bioproject accession	Biosample accession	Assembly accession	IMG OID
Corynebacterium singulare DSM 44357	Riegel et al. 1997 emend. Nouioui et al. 2018	CCUG 37330; JCM 10385; IFO 16162; NBRC 16162; CIP 105491; IBS B52218	*Corynebacterium singulare*	2830 499	60.1	2561	Gp0109683	PRJNA246651	SAMN03177398	GCA_000833575	
Corynebacterium testudinoris DSM 44614	Collins et al. 2001 emend. Nouioui et al. 2018	CCUG 41823; JCM 12108; CIP 106763; M935/96/4	*Corynebacterium testudinoris*	2721 226	63.1	2560	Gp0114695	PRJNA280910	SAMN03480629	GCA_001021045	
Corynebacterium ureicelerivorans DSM 45051	Yassin 2007 emend. Nouioui et al. 2018	CCUG 53377; JCM 15295; IMMIB RIV-2301	*Corynebacterium ureicelerivorans*	2328 188	65.0	1922	Gp0103378	PRJNA257688	SAMN02953970	GCA_000747315	
Corynebacterium deserti DSM 45689	Zhou et al. 2012 emend. Nouioui et al. 2018	NRRL B-59552; CCTCC AB 2010341; GIMN1.010	*Corynebacterium deserti*	3033 881	55.3	2724	Gp0109766	PRJNA222609	SAMN02950576	GCA_001277995	
Corynebacterium glyciniphilum ATCC 21341	(ex Kubota et al. 1972) Al-Dilaimi et al. 2015	AJ 3170; DSM 45795	*Corynebacterium glyciniphilum*	3568 218	64.8	3270	Gp0047724	PRJNA221205	SAMN03081498	GCA_000626675	2576861442
Corynebacterium epidermidicanis DSM 45586	Frischmann et al. 2012 emend. Nouioui et al. 2018	410; LMG 26322; CCUG 60915	*Corynebacterium epidermidicanis*	2692 072	58.1	2465	Gp0114694	PRJNA280479	SAMN03462986	GCA_001021025	
Corynebacterium minutissimum ATCC 23348	(ex Sarkany et al. 1962) Collins and Jones 1983 emend. Nouioui et al. 2018	CCUG 541; DSM 20651; JCM 9387; ATCC 23348; IFO 15361; NBRC 15361; NCTC 10288; CIP 100652	*Corynebacterium minutissimum*	2663 401	60.0	2276	Gp0122002	PRJNA264738	SAMN03140311	GCA_000805675	

Strain	Authority	Other deposits	Synonyms	Base pairs	Percent G+C	No. proteins	Goldstamp	Bioproject accession	Biosample accession	Assembly accession	IMG OID
Corynebacterium minutissimum NBRC 15361	(ex Sarkany et al. 1962) Collins and Jones 1983 emend. Nouioui et al. 2018	CCUG 541; DSM 20651; JCM 9387; ATCC 23348; IFO 15361; NBRC 15361; NCTC 10288; CIP 100652	*Corynebacterium minutissimum*	2663 455	60.0	2464	Gp0024448	PRJDB438	SAMD00046517	GCA_001552395	
Corynebacterium halotolerans YIM 70093	Chen et al. 2004 emend. Nouioui et al. 2018	DSM 44683; JCM 12676; CCTCC AA 001024; YIM 70093	*Corynebacterium halotolerans*	3222 002	68.3	2865	Gp0023456	PRJNA168616	SAMN02603027	GCA_000341345	2524023198
Corynebacterium xerosis NBRC 16721	(Lehmann and Neumann 1896) Lehmann and Neumann 1899 emend. Nouioui et al. 2018	CCUG 27544; DSM 20743; JCM 1971; ATCC 373; IFO 16721; NBRC 16721; NCTC 11861; CIP 100653	*Bacillus xerosis*; *Corynebacterium xerosis*	2686 219	69.7	2351	Gp0024449	PRJDB439	SAMD00046521	GCA_001552415	
Corynebacterium nuruki S6-4	Shin et al. 2011 emend. Nouioui et al. 2018	DSM 45695; JCM 17162; KACC 15032	*Corynebacterium nuruki*	3106 595	69.5	2787	Gp0011122	PRJNA66913	SAMN02470217	GCA_000213935	2547132106
Corynebacterium glutamicum ATCC 13032	(Kinoshita et al. 1958) Abe et al. 1967 emend. Nouioui et al. 2018	LMG 3730; NRRL B-2784; CCUG 27702; DSM 20300; JCM 1318; IFO 12168; NBRC 12168; CIP 82.08; HAMBI 2052	*Corynebacterium glutamicum*; *Micrococcus glutamicus*	3282 708	53.8	3057	Gp0000615	PRJNA13760	SAMEA3138338	GCA_000196335	639279306

Strain	Authority	Other deposits	Synonyms	Base pairs	Percent G+C	No. proteins	Goldstamp	Bioproject accession	Biosample accession	Assembly accession	IMG OID
Corynebacterium urealyticum DSM 7109	Pitcher et al. 1992 emend. Nouioui et al. 2018	LMG 19041; CCUG 18158; DSM 7109; JCM 10395; ATCC 43042; NCTC 12011; CIP 103524	*Corynebacterium urealyticum*	2369 219	64.2	2024	Gp0001357	PRJNA29211	SAMEA3138282	GCA_000069945	641522620
Corynebacterium marinum CGMCC 1.6998	Du et al. 2010 emend. Nouioui et al. 2018	7015; NRRL B-24779; DSM 44953; CGMCC 1.6998; D7015	*Corynebacterium marinum*	2680 020	67.9	2569		PRJDB10509	SAMD00245145	GCA_014645275	
Propionibacterium cyclohexanicum DSM 16859	Kusano et al. 1997	CCUG 48885; NRIC 247; JCM 21245; ATCC 700429; NBRC 103082; CIP 105414; IAM 14535; TA-12	*Propionibacterium cyclohexanicum*	2808 867	66.9	2525	Gp0131643	PRJNA332060	SAMN05443377	GCA_900111365	2675903216
Dermabacter vaginalis AD1-86	Chang et al. 2016	KCTC 39585; DSM 100050	*Dermabacter vaginalis*	2392 314	62.6	2129	Gp0203766	PRJNA286956	SAMN03774729	GCA_001678905	
Corynebacterium pacaense Marseille-P2417 T	Bellali et al. 2019	CSUR P2417	*Corynebacterium pacaense*	3027 822	63.7	2736		PRJEB19973	SAMEA103910525	GCA_900169525	
Corynebacterium oculi NML 130210	Bernard et al. 2016	LMG 28277; CCUG 65816; R-50187; TVRM83/2006F4/44	*Corynebacterium oculi*	2413 873	64.8	2327	Gp0145072	PRJNA295863	SAMN04091236	GCA_001412105	

Strain	Authority	Other deposits	Synonyms	Base pairs	Percent G+C	No. proteins	Goldstamp	Bioproject accession	Biosample accession	Assembly accession	IMG OID
Corynebacterium gottingense DSM 103494	Atasayar et al. 2017 emend. Bernard et al. 2020	22991/2016; 99221/2016; JCM 31931	*Corynebacterium gottingense*	2616814	65.5	2362	Gp0377217	PRJNA497573	SAMN10261082	GCA_003693265	
Corynebacterium timonense 5401744	Merhej et al. 2009 emend. Nouioui et al. 2018	5401744; CCUG 53856; DSM 45434; CIP 109424; CSUR P20	*Corynebacterium timonense*	2551022	66.9	2376	Gp0023376	PRJEB67	SAMEA2271986	GCA_000312345	2551306128
Corynebacterium pilosum CIP 103422	Yanagawa and Honda 1978	CCUG 27193; DSM 20521; JCM 3714; ATCC 29592; IFO 15285; NBRC 15285; NCTC 11862; CIP 103422	*Corynebacterium pilosum*	2545970	60.7	2772	Gp0120674	PRJNA284680	SAMN03731012	GCA_001044155	
Corynebacterium dentalis Marseille-P4122	Benabdelkader et al. 2020	CSURP4122	*Corynebacterium dentalis*	2302937	59.9	2080		PRJNA224116	SAMEA104348950	GCF_900232865	
Corynebacterium lowii LMG 28276	Bernard et al. 2016	CCUG 65815; NML 130206; R-50085; TKD4	*Corynebacterium lowii*	2354433	62.9	2108		PRJNA224116	SAMN04091594	GCF_001412085	
Corynebacterium liangguodongii 2184	Zhu et al. 2020	DSM 106203; CGMCC 1.16417	*Corynebacterium liangguodongii*	2357924	66.1	2115		PRJNA431327	SAMN08388739	GCA_003070865	
Corynebacterium jeikeium ATCC 43734	Jackman et al. 1988 emend. Nouioui et al. 2018	CCUG 27192; DSM 46361; DSM 7171; JCM 9384; ATCC 43734; NCTC 11913; CIP 103337	*Corynebacterium jeikeium*	2425907	61.6	2224	Gp0004510	PRJNA31445	SAMN00001506	GCA_000163435	647000231

Strain	Authority	Other deposits	Synonyms	Base pairs	Percent G+C	No. proteins	Goldstamp	Bioproject accession	Biosample accession	Assembly accession	IMG OID
Corynebacterium neomassiliense Marseille-P3888	Boxberger et al. 2020	CCUG72352; CSURP3888	*Corynebacterium neomassiliense*	3139653	66.9	2729		PRJNA224116	SAMEA5140071	GCF_900626215	
Corynebacterium diphtheriae NCTC 11397	(Kruse 1886) Lehmann and Neumann 1896 emend. Nouioui et al. 2018	DSM 44123; ATCC 27010; NCTC 11397; CIP 100721	*Bacillus diphtheriae*; *Corynebacterium diphtheriae*; *Corynebacterium diphtheriae* subsp. *diphtheriae*	2463666	53.5	2337	Gp0132011	PRJEB6403	SAMEA2517360	GCA_001457455	
Corynebacterium argentoratense DSM 44202	Riegel et al. 1995 emend. Nouioui et al. 2018	CCUG 34893; JCM 10392; ATCC 51927; CIP 104296; IBS B10697	*Corynebacterium argentoratense*	2031862	58.9	1875	Gp0044215	PRJNA209048	SAMN02603032	GCA_000590555	2554235426
Corynebacterium terpenotabidum Y-11	Takeuchi et al. 1999	DSM 44721; JCM 10555; IFO 14764; NBRC 14764; VKM Ac-2071; CIP 105927	*Corynebacterium terpenotabidum*	2751233	67.0	2369	Gp0022753	PRJNA168617	SAMN02603028	GCA_000418365	2554235357
Cargentoratense_DSM44202.fna				2031902	58.9	1896					
Catypicum_R2070.fna				2359448	65.4	2165					
Caurimucosum_ATCC700975.fna				2819226	60.6	2662					
Cbelfantii_FRC0043.fna				2609417	53.6	2663					
Ccallunae_DSM20147.fna				2928683	52.5	2707					
Ccamporealensis_DSM44610.fna				2451810	59.4	2263					

Strain	Authority	Other deposits	Synonyms	Base pairs	Percent G+C	No. proteins	Goldstamp	Bioproject accession	Biosample accession	Assembly accession	IMG OID
Ccasei_LMGS19264.fna				3132213	55.7	2889					
Cdeserti_GIMN1010.fna				3033893	55.3	2818					
Cdiphtheriae_NCTC11397.fna				2463666	53.5	2343					
Cdoosanense_DSM45436.fna				2698995	66.8	2609					
Cefficiens_YS314.fna				3219505	63.0	2877					
Cepidermidicanis_DSM45586.fna				2692072	58.1	2485					
Cfalsenii_DSM44353.fna				2719616	63.2	2371					
Cglutamicum_ATCC13032.fna				3282708	53.8	3031					
Cglycinophilum_AJ3170.fna				3568218	64.8	3325					
Chalotolerans_YIM70093.fna				3222008	68.3	2908					
Chumireducens_DSM45392.fna				2681312	68.6	2586					
Cimitans_DSM44264.fna				2565321	64.3	2367					
Cjeikeium_K41.fna				2476822	61.4	2137					
Ckroppenstedtii_DSM44385.fna				2446804	57.5	2127					

Methods, Results and References

The genome sequence data were uploaded to the Type (Strain) Genome Server (TYGS), a free bioinformatics platform available under https://tygs.dsmz.de, for a whole genome-based taxonomic analysis [1]. The results were provided by the TYGS on 2021-02-09. The TYGS analysis was subdivided into the following steps:

Determination of closely related type strains

Determination of closest type strain genomes was done in two complementary ways: First, all user genomes were compared against all type strain genomes available in the TYGS database via the MASH algorithm, a fast approximation of intergenomic relatedness [2], and, the ten type strains with the smallest MASH distances chosen per user genome. Second, an additional set of ten closely related type strains was determined via the 16S rDNA gene sequences. These were extracted from the user genomes using RNAmmer [3] and each sequence was subsequently BLASTed [4] against the 16S rDNA gene sequence of each of the currently 14130 type strains available in the TYGS database. This was used as a proxy to find the best 50 matching type strains (according to the bitscore) for each user genome and to subsequently calculate precise distances using the Genome BLAST Distance Phylogeny approach (GBDP) under the algorithm 'coverage' and distance formula d_5 [5]. These distances were finally used to determine the 10 closest type strain genomes for each of the user genomes.

Pairwise comparison of genome sequences

For the phylogenomic inference, all pairwise comparisons among the set of genomes were conducted using GBDP and accurate intergenomic distances inferred under the algorithm 'trimming' and distance formula d_5 [5]. 100 distance replicates were calculated each. Digital DDH values and confidence intervals were calculated using the recommended settings of the GGDC 2.1 [5].

Phylogenetic inference

The resulting intergenomic distances were used to infer a balanced minimum evolution tree with branch support via FASTME 2.1.4 including SPR postprocessing [6]. Branch support was inferred from 100 pseudo-bootstrap replicates each. The trees were rooted at the midpoint [7] and visualized with PhyD3 [8].

Type-based species and subspecies clustering

The type-based species clustering using a 70% dDDH radius around each of the 120 type strains was done as previously described [1]. The resulting groups are shown in Table 1 and 4. Subspecies clustering was done using a 79% dDDH threshold as previously introduced [9].

Results

Type-based species and subspecies clustering

The resulting species and subspecies clusters are listed in Table 4, whereas the taxonomic identification of the query strains is found in Table 1. Briefly, the clustering yielded 94 species clusters and the provided query strains were assigned to 20 of these. Moreover, user strains were located in 20 of 98 subspecies clusters.

Figure caption SSU tree

Figure 1. Tree inferred with FastME 2.1.6.1 [6] from GBDP distances calculated from 16S rDNA gene sequences. The branch lengths are scaled in terms of GBDP distance formula d_5. The numbers above branches are GBDP pseudo-bootstrap support values > 60 % from 100 replications, with an average branch support of 72.7 %. The tree was rooted at the midpoint [7].

Figure caption genome tree

Figure 2. Tree inferred with FastME 2.1.6.1 [6] from GBDP distances calculated from genome sequences. The branch lengths are scaled in terms of GBDP distance formula d_5. The numbers above branches are GBDP pseudo-bootstrap support values > 60 % from 100 replications, with an average branch support of 38.4 %. The tree was rooted at the midpoint [7].

References

[1] Meier-Kolthoff JP, Göker M. TYGS is an automated high-throughput platform for state-of-the-art genome-based taxonomy. Nat. Commun. 2019;10: 2182. DOI: 10.1038/s41467-019-10210-3

[2] Ondov BD, Treangen TJ, Melsted P, et al. Mash: Fast genome and metagenome distance estimation using MinHash. Genome Biol 2016;17: 1–14. DOI: 10.1186/s13059-016-0997-x

[3] Lagesen K, Hallin P. RNAmmer: consistent and rapid annotation of ribosomal RNA genes. Nucleic Acids Res. Oxford Univ Press; 2007;35: 3100–3108. DOI: 10.1093/nar/gkm160

[4] Camacho C, Coulouris G, Avagyan V, Ma N, Papadopoulos J, Bealer K, et al. BLAST+: architecture and applications. BMC Bioinformatics. 2009;10: 421. DOI: 10.1186/1471-2105-10-421

[5] Meier-Kolthoff JP, Auch AF, Klenk H-P, Göker M. Genome sequence-based species delimitation with confidence intervals and improved distance functions. BMC Bioinformatics. 2013;14: 60. DOI: 10.1186/1471-2105-14-60

[6] Lefort V, Desper R, Gascuel O. FastME 2.0: A comprehensive, accurate, and fast distance-based phylogeny inference program. Mol Biol Evol. 2015;32: 2798–2800. DOI: 10.1093/molbev/msv150

[7] Farris JS. Estimating phylogenetic trees from distance matrices. Am Nat. 1972;106: 645–667.

[8] Kreft L, Botzki A, Coppens F, Vandepoele K, Van Bel M. PhyD3: A phylogenetic tree viewer with extended phyloXML support for functional genomics data visualization. Bioinformatics. 2017;33: 2946–2947. DOI: 10.1093/bioinformatics/btx324

[9] Meier-Kolthoff JP, Hahnke RL, Petersen J, Scheuner C, Michael V, Fiebig A, et al. Complete genome sequence of DSM 30083^T, the type strain (U5/41^T) of *Escherichia coli*, and a proposal for delineating subspecies in microbial taxonomy. Stand Genomic Sci. 2014;9: 2. DOI: 10.1186/1944-3277-9-2

TYPE (STRAIN) GENOME SERVER

JOB SUMMARY

PRINT DATE: 2021-02-09 14:54:06 +0100

JOB ID: 8b24fa74-25ea-4add-bb8c-3a2924f86616

RESULT PAGE: https://tygs.dsmz.de/user_results/show?guid=8b24fa74-25ea-4add-bb8c-3a2924f86616

Table 1: Phylogenies

Publication-ready versions of both the genome-scale GBDP tree and the 16S rRNA gene sequence tree can be customized and exported either in SVG (vector graphic) or PNG format from within the phylogeny viewers in your TYGS result page. For publications the **SVG format is recommended** because it is lossless, always keeps its high resolution and can also be easily converted to other popular formats such as PDF or EPS. Please follow the link provided above!

Table 2: Identification

The below list contains the result of the TYGS species identification routine.

Explanation of remarks that might occur in the below table:

remark [R1]: The TYGS type strain database is automatically updated on an almost daily basis. However, if a particular type strain genome is not available in the TYGS database, this can have several reasons which are detailed in the FAQ. You can request an extended 16S rRNA gene analysis via the 16S tree viewer found in your result page to detect **not yet genome-sequenced** type strains relevant for your study.

remark [R2]: > 70% dDDH value (formula d_4) and (almost) minimal dDDH values for gene-content formulae d_0 and d_6 indicate a potentially unreliable identification result and should thus be checked via the 16S rRNA gene sequence similarity. Such strong deviations can, in principle, be caused by sequence contamination.

remark [R3]: G+C content difference of > 1 % indicates a potentially unreliable identification result because within species G+C content varies no more than 1 %, if computed from genome sequences (PMID: 24505073).

Strain	Conclusion	Identification result	Remark
**'Cvitaeruminis_DSM20294'**	belongs to known species	*Corynebacterium vitaeruminis*	
**'CriegeliiPUDD83A45'**	belongs to known species	*Corynebacterium riegelii*	
**'Cmaris_DSM45190'**	belongs to known species	*Corynebacterium maris*	
**'Csilvaticum_KL0182'**	belongs to known species	*Corynebacterium silvaticum*	
**'Curealyticum_DSM7109'**	belongs to known species	*Corynebacterium urealyticum*	
**'Crouxii_FRC0190'**	belongs to known species	*Corynebacterium rouxii*	
**'Cpseudotuberculosis_31'**	belongs to known species	*Corynebacterium pseudotuberculosis*	
**'Cpseudotuberculosis_ATCC19410'**	belongs to known species	*Corynebacterium pseudotuberculosis*	
**'Culcerans_NCTC7910'**	belongs to known species	*Corynebacterium ulcerans*	
**'Ckutscheri_DSM20755'**	belongs to known species	*Corynebacterium kutscheri*	
**'Cmarinum_DSM44953'**	belongs to known species	*Corynebacterium marinum*	
**'Cmustelae_DSM45274'**	belongs to known species	*Corynebacterium mustelae*	
**'Csingulare_IBSB52218'**	belongs to known species	*Corynebacterium singulare*	
**'Ctestudinoris_DSM44614'**	belongs to known species	*Corynebacterium testudinoris*	
**'Cureicelerivorans_IMMIBRIV2301'**	belongs to known species	*Corynebacterium ureicelerivorans*	

Strain	Conclusion	Identification result	Remark
**'Cuterequi_DSM45634'**	belongs to known species	*Corynebacterium uterequi*	
**'Clactis_RW25'**	belongs to known species	*Corynebacterium lactis*	
**'Cterpenotabidum_Y11'**	belongs to known species	*Corynebacterium terpenotabidum*	
**'Csimulans_PES1'**	potential new species		see [R1]
**'Culcerans_NCTC12077'**	potential new species		see [R1]

Table 3: Pairwise comparisons of user genomes vs. type-strain genomes

The overall number of pairwise comparisons was too large for a proper display and was thus reduced to only those comparisons having a digital DDH value ≥ 65 % in at least one of the three formulae d_0, d_4 and d_6.

The following table contains the pairwise dDDH values between your user genomes and the selected type-strain genomes. The dDDH values are provided along with their confidence intervals (C.I.) for the three different GBDP formulas:

- formula d_0 (a.k.a. GGDC formula 1): length of all HSPs divided by total genome length
- formula d_4 (a.k.a. GGDC formula 2): sum of all identities found in HSPs divided by overall HSP length
- formula d_6 (a.k.a. GGDC formula 3): sum of all identities found in HSPs divided by total genome length

Note: Formula d_4 is independent of genome length and is thus robust against the use of incomplete draft genomes. For other reasons for preferring formula d_4, see the FAQ.

Query	Subject	d_0	C.I. d_0	d_4	C.I. d_4	d_6	C.I. d_6	Diff. G+C Percent
**'Cterpenotabidum_Y11.fna'**	*Corynebacterium terpenotabidum* Y-11	100.0	[100.0 - 100.0]	100.0	[100.0 - 100.0]	100.0	[100.0 - 100.0]	0.0
**'Cmaris_DSM45190.fna'**	*Corynebacterium maris* DSM 45190	100.0	[100.0 - 100.0]	100.0	[100.0 - 100.0]	100.0	[100.0 - 100.0]	0.0
**'Culcerans_NCTC7910.fna'**	*Corynebacterium ulcerans* NCTC 7910	100.0	[100.0 - 100.0]	100.0	[100.0 - 100.0]	100.0	[100.0 - 100.0]	0.0
**'Cmarinum_DSM44953.fna'**	*Corynebacterium marinum* DSM 44953	100.0	[100.0 - 100.0]	100.0	[100.0 - 100.0]	100.0	[100.0 - 100.0]	0.0
**'Cureicelerivorans_IMMIBRIV2301.fna'**	*Corynebacterium ureicelerivorans* DSM 45051	100.0	[100.0 - 100.0]	100.0	[100.0 - 100.0]	100.0	[100.0 - 100.0]	0.0
**'Cmarinum_DSM44953.fna'**	*Corynebacterium marinum* CGMCC 1.6998	99.9	[99.8 - 100.0]	100.0	[100.0 - 100.0]	100.0	[99.9 - 100.0]	0.2
**'Csilvaticum_KL0182.fna'**	*Corynebacterium silvaticum* KL0182	100.0	[100.0 - 100.0]	100.0	[100.0 - 100.0]	100.0	[100.0 - 100.0]	0.01
**'Csingulare_IBSB52218.fna'**	*Corynebacterium singulare* DSM 44357	100.0	[100.0 - 100.0]	100.0	[100.0 - 100.0]	100.0	[100.0 - 100.0]	0.0
**'Ctestudinoris_DSM44614.fna'**	*Corynebacterium testudinoris* DSM 44614	100.0	[100.0 - 100.0]	100.0	[100.0 - 100.0]	100.0	[100.0 - 100.0]	0.0
**'Curealyticum_DSM7109.fna'**	*Corynebacterium urealyticum* DSM 7109	100.0	[100.0 - 100.0]	100.0	[100.0 - 100.0]	100.0	[100.0 - 100.0]	0.0
**'Cpseudotuberculosis_ATCC19410.fna'**	*Corynebacterium pseudotuberculosis* ATCC 19410	100.0	[100.0 - 100.0]	100.0	[100.0 - 100.0]	100.0	[100.0 - 100.0]	0.0
**'Clactis_RW25.fna'**	*Corynebacterium lactis* DSM 45799	100.0	[100.0 - 100.0]	100.0	[100.0 - 100.0]	100.0	[100.0 - 100.0]	0.0
**'Cmustelae_DSM45274.fna'**	*Corynebacterium mustelae* DSM 45274	100.0	[100.0 - 100.0]	100.0	[100.0 - 100.0]	100.0	[100.0 - 100.0]	0.0
**'Cpseudotuberculosis_ATCC19410.fna'**	*Corynebacterium pseudotuberculosis* DSM 20689	100.0	[100.0 - 100.0]	100.0	[100.0 - 100.0]	100.0	[100.0 - 100.0]	0.0
**'Cvitaeruminis_DSM20294.fna'**	*Corynebacterium vitaeruminis* DSM 20294	100.0	[100.0 - 100.0]	100.0	[100.0 - 100.0]	100.0	[100.0 - 100.0]	0.0
**'Curealyticum_DSM7109.fna'**	*Corynebacterium urealyticum* NCTC12011	100.0	[100.0 - 100.0]	100.0	[100.0 - 100.0]	100.0	[100.0 - 100.0]	0.02
**'Ckutscheri_DSM20755.fna'**	*Corynebacterium kutscheri* NCTC 11138	100.0	[100.0 - 100.0]	100.0	[99.9 - 100.0]	100.0	[100.0 - 100.0]	0.0
**'Ckutscheri_DSM20755.fna'**	*Corynebacterium kutscheri* DSM 20755	100.0	[100.0 - 100.0]	100.0	[100.0 - 100.0]	100.0	[100.0 - 100.0]	0.0
**'Crouxii_FRC0190.fna'**	*Corynebacterium rouxii* FRC0190 T	100.0	[100.0 - 100.0]	100.0	[100.0 - 100.0]	100.0	[100.0 - 100.0]	0.0
**'Cuterequi_DSM45634.fna'**	*Corynebacterium uterequi* DSM 45634	100.0	[100.0 - 100.0]	100.0	[100.0 - 100.0]	100.0	[100.0 - 100.0]	0.0

Query	Subject	d_0	C.I. d_0	d_4	C.I. d_4	d_6	C.I. d_6	Diff. G+C Percent
**'Cpseudotuberculosis_31.fna'**	*Corynebacterium pseudotuberculosis* ATCC 19410	99.1	[98.3 - 99.5]	89.4	[87.0 - 91.4]	99.0	[98.3 - 99.4]	0.02
**'Cpseudotuberculosis_31.fna'**	*Corynebacterium pseudotuberculosis* DSM 20689	99.1	[98.3 - 99.5]	89.4	[87.0 - 91.3]	99.0	[98.3 - 99.4]	0.02
**'Cpseudotuberculosis_31.fna'**	**'Cpseudotuberculosis_ATCC19410.fna'**	99.1	[98.3 - 99.5]	89.4	[87.0 - 91.4]	99.0	[98.3 - 99.4]	0.02
**'CriegeliiPUDD83A45.fna'**	*Corynebacterium riegelii* DSM 44326	92.0	[89.1 - 94.2]	71.9	[68.9 - 74.7]	91.3	[88.7 - 93.4]	0.11
**'Culcerans_NCTC12077.fna'**	*Corynebacterium ulcerans* NCTC 7910	94.4	[92.0 - 96.2]	66.8	[63.8 - 69.6]	92.3	[89.9 - 94.2]	0.07
**'Culcerans_NCTC7910.fna'**	**'Culcerans_NCTC12077.fna'**	94.4	[92.0 - 96.2]	66.8	[63.8 - 69.6]	92.3	[89.9 - 94.2]	0.07
**'Cureicelerivorans_IMMIBRIV2301.fna'**	*Corynebacterium mucifaciens* ATCC 700355	78.9	[74.9 - 82.3]	65.7	[62.8 - 68.5]	79.1	[75.7 - 82.2]	0.48
**'Crouxii_FRC0190.fna'**	*Corynebacterium diphtheriae* NCTC 11397	75.7	[71.7 - 79.3]	49.3	[46.7 - 51.9]	71.8	[68.4 - 75.1]	0.3
**'Crouxii_FRC0190.fna'**	*Corynebacterium diphtheriae* DSM 44123	77.9	[73.9 - 81.4]	49.1	[46.5 - 51.7]	73.6	[70.1 - 76.8]	0.31
**'Crouxii_FRC0190.fna'**	*Corynebacterium belfantii* FRC0043	73.0	[69.0 - 76.6]	45.4	[42.8 - 47.9]	68.2	[64.8 - 71.4]	0.4
**'Csilvaticum_KL0182.fna'**	**'Culcerans_NCTC12077.fna'**	87.1	[83.5 - 90.0]	41.0	[38.5 - 43.6]	77.6	[74.1 - 80.7]	1.06
**'Culcerans_NCTC12077.fna'**	*Corynebacterium silvaticum* KL0182	87.1	[83.5 - 89.9]	41.0	[38.5 - 43.6]	77.6	[74.1 - 80.7]	1.06
**'Culcerans_NCTC7910.fna'**	*Corynebacterium silvaticum* KL0182	91.7	[88.7 - 94.0]	40.9	[38.4 - 43.5]	81.5	[78.1 - 84.4]	1.14
**'Csilvaticum_KL0182.fna'**	**'Culcerans_NCTC7910.fna'**	91.7	[88.7 - 94.0]	40.9	[38.4 - 43.5]	81.5	[78.2 - 84.5]	1.13
**'Csilvaticum_KL0182.fna'**	*Corynebacterium ulcerans* NCTC 7910	91.7	[88.7 - 94.0]	40.9	[38.4 - 43.5]	81.5	[78.2 - 84.5]	1.13
**'Csilvaticum_KL0182.fna'**	*Corynebacterium pseudotuberculosis* DSM 20689	82.9	[79.1 - 86.2]	28.6	[26.2 - 31.0]	65.9	[62.6 - 69.2]	2.26
**'Cpseudotuberculosis_31.fna'**	**'Csilvaticum_KL0182.fna'**	83.5	[79.7 - 86.7]	28.5	[26.1 - 31.0]	66.3	[62.9 - 69.6]	2.28
**'Csilvaticum_KL0182.fna'**	*Corynebacterium pseudotuberculosis* ATCC 19410	82.8	[78.9 - 86.1]	28.5	[26.2 - 31.0]	65.9	[62.5 - 69.1]	2.26
**'Cpseudotuberculosis_31.fna'**	*Corynebacterium silvaticum* KL0182	83.5	[79.7 - 86.7]	28.5	[26.1 - 31.0]	66.3	[62.9 - 69.5]	2.28
**'Cpseudotuberculosis_ATCC19410.fna'**	**'Csilvaticum_KL0182.fna'**	82.8	[78.9 - 86.1]	28.5	[26.2 - 31.0]	65.9	[62.5 - 69.1]	2.26
**'Cpseudotuberculosis_ATCC19410.fna'**	*Corynebacterium silvaticum* KL0182	82.8	[78.9 - 86.0]	28.5	[26.2 - 31.0]	65.8	[62.5 - 69.1]	2.27
**'Csingulare_IBSB52218.fna'**	*Corynebacterium minutissimum* NCTC 10289	67.3	[63.4 - 71.0]	27.8	[25.4 - 30.2]	54.8	[51.6 - 57.9]	0.17
**'Cpseudotuberculosis_ATCC19410.fna'**	**'Culcerans_NCTC7910.fna'**	88.1	[84.6 - 90.8]	27.7	[25.3 - 30.2]	68.9	[65.5 - 72.2]	1.13
**'Cpseudotuberculosis_31.fna'**	*Corynebacterium ulcerans* NCTC 7910	87.2	[83.7 - 90.1]	27.7	[25.3 - 30.2]	68.3	[64.8 - 71.5]	1.15
**'Cpseudotuberculosis_ATCC19410.fna'**	*Corynebacterium ulcerans* NCTC 7910	88.1	[84.6 - 90.8]	27.7	[25.3 - 30.2]	68.9	[65.5 - 72.2]	1.13

Query	Subject	d_0	C.I. d_0	d_4	C.I. d_4	d_6	C.I. d_6	Diff. G+C Percent
**'Culcerans_NCTC7910.fna'**	*Corynebacterium pseudotuberculosis* DSM 20689	88.1	[84.6 - 90.8]	27.7	[25.3 - 30.2]	68.9	[65.5 - 72.2]	1.13
**'Culcerans_NCTC7910.fna'**	*Corynebacterium pseudotuberculosis* ATCC 19410	88.1	[84.6 - 90.8]	27.7	[25.3 - 30.2]	68.9	[65.5 - 72.2]	1.13
**'Cpseudotuberculosis_31.fna'**	**'Culcerans_NCTC7910.fna'**	87.2	[83.7 - 90.1]	27.7	[25.3 - 30.2]	68.3	[64.8 - 71.5]	1.15
**'Culcerans_NCTC12077.fna'**	*Corynebacterium pseudotuberculosis* DSM 20689	82.5	[78.6 - 85.8]	27.6	[25.2 - 30.1]	64.8	[61.4 - 68.0]	1.2
**'Cpseudotuberculosis_31.fna'**	**'Culcerans_NCTC12077.fna'**	82.3	[78.4 - 85.6]	27.6	[25.2 - 30.1]	64.6	[61.3 - 67.8]	1.22
**'Culcerans_NCTC12077.fna'**	*Corynebacterium pseudotuberculosis* ATCC 19410	82.5	[78.6 - 85.8]	27.6	[25.2 - 30.1]	64.8	[61.4 - 68.0]	1.2
**'Cpseudotuberculosis_ATCC19410.fna'**	**'Culcerans_NCTC12077.fna'**	82.5	[78.6 - 85.8]	27.6	[25.2 - 30.1]	64.8	[61.4 - 68.0]	1.2
**'Csingulare_IBSB52218.fna'**	*Corynebacterium minutissimum* NCTC10288	67.7	[63.9 - 71.4]	27.4	[25.0 - 29.8]	54.7	[51.6 - 57.8]	0.22
**'Csingulare_IBSB52218.fna'**	*Corynebacterium minutissimum* ATCC 23348	67.8	[63.9 - 71.5]	27.2	[24.8 - 29.7]	54.7	[51.5 - 57.8]	0.16
**'Csingulare_IBSB52218.fna'**	*Corynebacterium minutissimum* NBRC 15361	67.9	[64.0 - 71.5]	27.2	[24.9 - 29.7]	54.7	[51.6 - 57.8]	0.17

Table 4: Strains in your dataset

Joint dataset of automatically determined closest type strains (if this mode was chosen), manually selected type strains (if selected accordingly) and the provided user strains, if provided (marked in yellow).

Strain	Authority	Other deposits	Synonyms	Base pairs	Percent G+C	No. proteins	Goldstamp	Bioproject accession	Biosample accession	Assembly accession	IMG OID
Corynebacterium vitaeruminis DSM 20294	(Bechdel et al. 1928) Lanéelle et al. 1980	CCUG 28792; JCM 1323; ATCC 10234; IFO 12143; NBRC 12143; VKM B-1211; CIP 82.07; NCIB 9291; NCIMB 9291	*Brevibacterium vitaeruminis*; *Corynebacterium vitaeruminis*; *Flavobacterium vitarumen*	2931 780	65.5	2577	Gp0023683	PRJNA172966	SAMN03081455	GCA_000550805	2558860221
Corynebacterium falsenii DSM 44353	Sjödén et al. 1998 emend. Nouioui et al. 2018	CCUG 33651; JCM 11949; CIP 105466; Y13024	*Corynebacterium falsenii*	2719 559	63.2	2306	Gp0086746	PRJNA235944	SAMN02641485	GCA_000525655	2571042744
Corynebacterium sanguinis CCUG 58655T	Jaén-Luchoro et al. 2020	CCM 8873	*Corynebacterium sanguinis*	2362 885	65.3	2243		PRJNA224116	SAMN10573883	GCF_007641235	
Corynebacterium lubricantis DSM 45231	Kämpfer et al. 2009 emend. Nouioui et al. 2018	CCUG 56567; JCM 16607; CCM 7546; KSS-3Se	*Corynebacterium lubricantis*	2945 292	58.6	2818	Gp0013695	PRJNA165249	SAMN02256424	GCA_000379425	2515154018
Corynebacterium doosanense DSM 45436	Lee et al. 2009 emend. Nouioui et al. 2018	KCTC 19568; CCUG 57284; CAU 212	*Corynebacterium doosanense*	2649 019	66.9	2590	Gp0013691	PRJNA165377	SAMN02256506	GCA_000372245	2515154029
Corynebacterium ulceribovis DSM 45146	Yassin 2009 emend. Nouioui et al. 2018	CCUG 55727; IMMIB L-1395	*Corynebacterium ulceribovis*	2300 430	59.2	2104	Gp0013740	PRJNA165381	SAMN02256494	GCA_000372445	2515154059
Corynebacterium fournieri Marseille-P2948	Diop et al. 2018	DSM 103271; CSUR P2948	*Corynebacterium fournieri*	2357 034	65.0	2305	Gp0370410	PRJEB20393	SAMEA103975581	GCA_900176865	

Strain	Authority	Other deposits	Synonyms	Base pairs	Percent G+C	No. proteins	Goldstamp	Bioproject accession	Biosample accession	Assembly accession	IMG OID
Corynebacterium riegelii DSM 44326	Funke et al. 1998	CCUG 38180; JCM 10389; ATCC 700782; CIP 105310; DMMZ 2415	*Corynebacterium riegelii*	2519 232	60.4	2283		PRJNA231221	SAMN16357283		
Corynebacterium anserum 23H37-10	Liu et al. 2021	GDMCC 1.1737; KACC 21672	*Corynebacterium anserum*	2208 656	55.2	1764		PRJNA595090	SAMN13546099	GCA_014262665	
Corynebacterium godavarianum LMG 29598	Jani et al. 2018	MCC 3388; KCTC 39803; PRD07	*Corynebacterium godavarianum*	2521 298	65.6	2235		PRJNA555895	SAMN12335367	GCA_007559235	
Corynebacterium endometrii LMM-1653T	Ballas et al. 2020	LMG-31164; CCM 8952	*Corynebacterium endometrii*	2477 061	60.9	2189		PRJNA224116	SAMN11357123	GCF_004795735	
Corynebacterium aurimucosum strain DSM 44532	Yassin et al. 2002 emend. Daneshvar et al. 2004	NRRL B-24143; CCUG 47449; JCM 11766; IMMIB D-1488	*Corynebacterium aurimucosum*	2737 787	60.3	2417		PRJNA231221	SAMN16357278	GCA_000626615	
Corynebacterium alimapuense CCUG 69366	Claverias et al. 2019	NCIMB 15118; VA37-3	*Corynebacterium alimapuense*	2281 535	57.1	2040	Gp0385727	PRJNA305687	SAMN08535650	GCA_003716585	
Corynebacterium phoceense MC1	Cresci et al. 2016	DSM 100570; CSUR P1905	*Corynebacterium phoceense*	2772 735	63.2	2701		PRJNA224116	SAMEA4059842	GCF_900092335	
Corynebacterium diphtheriae subsp. *lausannense* CHUV2995	Tagini et al. 2019	CCUG 72509; DSM 107520	*Corynebacterium diphtheriae* subsp. *lausannense*	3060 363	53.9	3145	Gp0442955	PRJEB24256	SAMEA104679569	GCA_900312965	
Corynebacterium belfantii FRC0043	Dazas et al. 2018	DSM 105776; CIP 111412	*Corynebacterium belfantii*	2598 827	53.6	2557	Gp0364753	PRJEB22103	SAMEA104208677	GCA_900205605	

Strain	Authority	Other deposits	Synonyms	Base pairs	Percent G+C	No. proteins	Goldstamp	Bioproject accession	Biosample accession	Assembly accession	IMG OID
Corynebacterium urogenitale DSM 108747	Ballas et al. 2020	LMG 31163; LMM-1652	*Corynebacterium urogenitale*	2351 892	59.9	2058		PRJNA224116	SAMN12924940	GCF_009026825	
Corynebacterium frankenforstense ST18	Wiertz et al. 2013 emend. Nouioui et al. 2018	CCUG 63371; DSM 45800	*Corynebacterium frankenforstense*	2604 152	71.5	1801	Gp0118685	PRJNA232093	SAMN02991553	GCA_001941485	
Corynebacterium sphenisci DSM 44792	Goyache et al. 2003 emend. Nouioui et al. 2018	CCUG 46398; CECT 5990; JCM 12270	*Corynebacterium sphenisci*	2594 799	74.7	1827	Gp0118687	PRJNA232092	SAMN02996499	GCA_001941505	
Corynebacterium aquilae S-613	Fernández-Garayzábal et al. 2003 emend. Nouioui et al. 2018	CCUG 46511; CECT 5993; DSM 44791; JCM 12268	*Corynebacterium aquilae*	2926 436	60.9	2013	Gp0118683	PRJNA230273	SAMN02996496	GCA_001941445	
Corynebacterium afermentans DSM 44280	Riegel et al. 1993 emend. Nouioui et al. 2018	CCUG 32103; JCM 10390; ATCC 51403; CIP 103499; LCDC 88199	*Corynebacterium afermentans*; *Corynebacterium afermentans* subsp. *afermentans*	2326 687	64.9	2171	Gp0131801	PRJEB18848	SAMN05421802	GCA_900156035	2681813557
Corynebacterium mooreparkense DSM 44702	Brennan et al. 2001 emend. Nouioui et al. 2018	LMG S-19265; JCM 12073; CIP 107183; DPC 5310; NCIMB 30131	*Corynebacterium mooreparkense*	3433 007	67.1	3039	Gp0008259	PRJNA50001	SAMN02603088	GCA_000179395	2511231114
Corynebacterium nigricans ATCC 700975	Shukla et al. 2004 emend. Nouioui et al. 2018	CCUG 48176; DSM 44827; JCM 12684; CIP 107346; CN-1	*Corynebacterium nigricans*	2819 226	60.6	2551	Gp0004919	PRJNA37279	SAMN02603064	GCA_000022905	643692018
Corynebacterium urinapleomorphum Marseille-P2799	Niang et al. 2019	DSM103272; CSURP2799	*Corynebacterium urinapleomorphum*	2259 535	63.4	2097		PRJEB18932	SAMEA47264668	GCA_900155535	

Strain	Authority	Other deposits	Synonyms	Base pairs	Percent G+C	No. proteins	Goldstamp	Bioproject accession	Biosample accession	Assembly accession	IMG OID
Corynebacterium maris DSM 45190	Ben-Dov et al. 2009	LMG 24561; JCM 17018; Coryn-1	*Corynebacterium maris*	2833 547	66.6	2584	Gp0023680	PRJNA172964	SAMN02603057	GCA_000442645	2561511185
Corynebacterium silvaticum KL0182	Dangel et al. 2020	LMG 31313; DSM 109166; CIP 111672	*Corynebacterium silvaticum*	2548 487	54.4	2017		PRJNA517029	SAMN10039578	GCA_004382825	
Corynebacterium resistens DSM 45100	Otsuka et al. 2005 emend. Nouioui et al. 2018	CCUG 50093; JCM 12819; GTC 2026; SICGH 158	*Corynebacterium resistens*	2601 311	57.1	2171	Gp0003835	PRJNA39683	SAMN02603065	GCA_000177535	650716029
Kocuria soli M5W7-7	Tuo et al. 2019	KCTC 49195; CGMCC 1.13744	*Kocuria soli*	2949 090	67.0	2529		PRJNA501788	SAMN10345596	GCA_003797835	
Corynebacterium senegalense Marseille-P4329	Ndiaye et al. 2019	CSURP432 9	*Corynebacterium senegalense*	2310 902	68.7	2173	Gp0359219	PRJEB24601	SAMEA4664250	GCA_900411315	
Corynebacterium haemomassilien se Marseille-Q3615	Boxberger et al. 2020	CSUR Q3615	*Corynebacterium haemomassilien se*	2578 128	65.3	2331		PRJNA646616	SAMN15548222	GCA_013978595	
Corynebacterium striatum NBRC 15291	(Chester 1901) Eberson 1918 emend. Nouioui et al. 2018	CCUG 27949; DSM 20668; JCM 9390; ATCC 6940; IFO 15291; NBRC 15291; NCTC 764; CIP 81.15	*Bacterium striatum*; *Corynebacterium striatum*	3106 742	59.1	2949		PRJDB8042	SAMD00169825	GCA_006538485	

Strain	Authority	Other deposits	Synonyms	Base pairs	Percent G+C	No. proteins	Goldstamp	Bioproject accession	Biosample accession	Assembly accession	IMG OID
Corynebacterium xerosis ATCC 373	(Lehmann and Neumann 1896) Lehmann and Neumann 1899 emend. Nouioui et al. 2018	CCUG 27544; DSM 20743; JCM 1971; ATCC 373; IFO 16721; NBRC 16721; NCTC 11861; CIP 100653	*Bacillus xerosis*; *Corynebacterium xerosis*	2661 590	69.5	1843		PRJNA280206	SAMN03458390	GCA_000988235	
Corynebacterium diphtheriae DSM 44123	(Kruse 1886) Lehmann and Neumann 1896 emend. Nouioui et al. 2018	DSM 44123; ATCC 27010; NCTC 11397; CIP 100721	*Bacillus diphtheriae*; *Corynebacterium diphtheriae*; *Corynebacterium diphtheriae* subsp. *diphtheriae*	2364 574	53.5	2116		PRJNA296455	SAMN04099181	GCA_001913265	
Corynebacterium kutscheri NCTC 11138	(Migula 1900) Bergey et al. 1925 emend. Nouioui et al. 2018	CCUG 27535; DSM 20755; JCM 9385; ATCC 15677; IFO 15288; NBRC 15288; NCTC 11138; CIP 103423	*Bacterium kutscheri*; *Corynebacterium kutscheri*	2354 887	46.5	2101		PRJEB6403	SAMEA4530649	GCA_900637605	
Corynebacterium minutissimum NCTC10288	(ex Sarkany et al. 1962) Collins and Jones 1983 emend. Nouioui et al. 2018	CCUG 541; DSM 20651; JCM 9387; ATCC 23348; IFO 15361; NBRC 15361; NCTC 10288; CIP 100652	*Corynebacterium minutissimum*	2695 970	59.9	2463		PRJEB6403	SAMEA4030732	GCA_900478045	

Strain	Authority	Other deposits	Synonyms	Base pairs	Percent G+C	No. proteins	Goldstamp	Bioproject accession	Biosample accession	Assembly accession	IMG OID
Corynebacterium imitans NCTC 13015	Funke et al. 1997 emend. Nouioui et al. 2018	2023; CCUG 36877; DSM 44264; JCM 10386; ATCC 700354; IFO 16163; NBRC 16163; NBRC 100416; NCTC 13015; CIP 105130	*Corynebacterium imitans*	2565 606	64.3	2347		PRJEB6403	SAMEA4535761	GCA_900187215	
Corynebacterium urealyticum NCTC12011	Pitcher et al. 1992 emend. Nouioui et al. 2018	LMG 19041; CCUG 18158; DSM 7109; JCM 10395; ATCC 43042; NCTC 12011; CIP 103524	*Corynebacterium urealyticum*	2377 532	64.2	2005		PRJEB6403	SAMEA4530651	GCA_900187235	
Corynebacterium jeikeium NCTC 11913	Jackman et al. 1988 emend. Nouioui et al. 2018	CCUG 27192; DSM 46361; DSM 7171; JCM 9384; ATCC 43734; NCTC 11913; CIP 103337	*Corynebacterium jeikeium*	2526 027	61.4	2220		PRJEB6403	SAMEA4017703	GCA_900461185	
Corynebacterium minutissimum NCTC 10289	(ex Sarkany et al. 1962) Collins and Jones 1983 emend. Nouioui et al. 2018	CCUG 541; DSM 20651; JCM 9387; ATCC 23348; IFO 15361; NBRC 15361; NCTC 10288; CIP 100652	*Corynebacterium minutissimum*	2736 326	59.9	2571		PRJEB6403	SAMEA104016176	GCA_900447475	

Strain	Authority	Other deposits	Synonyms	Base pairs	Percent G+C	No. proteins	Goldstamp	Bioproject accession	Biosample accession	Assembly accession	IMG OID
Corynebacterium pilosum NCTC 11862	Yanagawa and Honda 1978	CCUG 27193; DSM 20521; JCM 3714; ATCC 29592; IFO 15285; NBRC 15285; NCTC 11862; CIP 103422	*Corynebacterium pilosum*	2593 653	60.7	2398		PRJEB6403	SAMEA4530650	GCA_900447205	
Corynebacterium striatum NCTC 764	(Chester 1901) Eberson 1918 emend. Nouioui et al. 2018	CCUG 27949; DSM 20668; JCM 9390; ATCC 6940; IFO 15291; NBRC 15291; NCTC 764; CIP 81.15	*Bacterium striatum*; *Corynebacterium striatum*	2924 414	59.1	2730		PRJEB6403	SAMEA4521472	GCA_900447675	
Corynebacterium spheniscorum CCUG 45512	Goyache et al. 2003 emend. Nouioui et al. 2018	CCUG 45512; CECT 5986; DSM 44757; JCM 12271; PG 39	*Corynebacterium spheniscorum*	2456 378	57.5	2059		PRJNA563568	SAMN12771121	GCA_008693095	
Corynebacterium humireducens NBRC 106098	Wu et al. 2011 emend. Nouioui et al. 2018	DSM 45392; NBRC 106098; CGMCC 2452; MFC-5	*Corynebacterium humireducens*	2609 893	68.8	2525	Gp0023682	PRJDB440	SAMD00046477	GCA_001571025	
Corynebacterium pseudopelargi CCM 8832	Busse et al. 2019	812CH; LMG 30627; CCUG 72167	*Corynebacterium pseudopelargi*	2348 160	57.9	2199	Gp0379416	PRJNA224116	SAMN08449372	GCF_003814005	
Corynebacterium tuscaniense CCUG 51321	Riegel et al. 2006	DSM 45101; JCM 15294; ATCC BAA-1141; ISS-5309	*Corynebacterium tuscaniense*	2232 117	59.4	2073		PRJNA224116	SAMN12771122	GCF_008693065	

Strain	Authority	Other deposits	Synonyms	Base pairs	Percent G+C	No. proteins	Goldstamp	Bioproject accession	Biosample accession	Assembly accession	IMG OID
Corynebacterium halotolerans DSM 44683	Chen et al. 2004 emend. Nouioui et al. 2018	DSM 44683; JCM 12676; CCTCC AA 001024; YIM 70093	*Corynebacterium halotolerans*	3202 499	68.4	2914	Gp0013694	PRJNA215338	SAMN02743911	GCA_000688435	2556921045
Corynebacterium variabile NBRC 15286	(Müller 1961) Collins 1987	NRRL B-4201; CCUG 45246; DSM 20132; JCM 2154; ATCC 15753; IFO 15286; VKM Ac-1122; CIP 102112; HAMBI 1872; NCIB 9455; NCIMB 9455	*Arthrobacter variabilis*; *Corynebacterium variabile*	3170 194	67.4	2983		PRJDB6001	SAMD00097555	GCA_006539825	
Corynebacterium tapiri LMG 28165	Baumgardt et al. 2015	2385/12; CCUG 65456	*Corynebacterium tapiri*	2246 156	62.3	2045		PRJNA545602	SAMN11928016	GCA_006334925	
Corynebacterium rouxii FRC0190 T	Badell et al. 2020	DSM 110354; CIP 111752	*Corynebacterium rouxii*	2451 019	53.2	2365		PRJNA224116	SAMEA5992727	GCF_902702935	
Corynebacterium mucifaciens ATCC 700355	Funke et al. 1997	CCUG 36878; DSM 44265; JCM 10384; CIP 105129; DMMZ 2278	*Corynebacterium mucifaciens*	2180 241	65.5	2028		PRJNA622446	SAMN14517857	GCA_012396315	
Corynebacterium spheniscorum DSM 44757	Goyache et al. 2003 emend. Nouioui et al. 2018	CCUG 45512; CECT 5986; DSM 44757; JCM 12271; PG 39	*Corynebacterium spheniscorum*	2451 870	57.5	2156	Gp0102878	PRJNA262359	SAMN05660282	GCA_900113445	2599185219

Strain	Authority	Other deposits	Synonyms	Base pairs	Percent G+C	No. proteins	Goldstamp	Bioproject accession	Biosample accession	Assembly accession	IMG OID
Corynebacterium pelargi DSM 46737	Kämpfer et al. 2015	136/3; LMG 28174; DSM 46737; CCM 8517; CIP 110778	*Corynebacterium pelargi*	2370060	58.2	2169	Gp0442046	PRJNA224116	SAMN06041739	GCA_004114895	
Corynebacterium hadale NBT06-6	Wei et al. 2018	MCCC 1K03347; DSM 105365	*Corynebacterium hadale*	2679199	65.2	2362	Gp0374689	PRJNA396693	SAMN07460149	GCA_002273005	
Corynebacterium pollutisoli VDS	Negi et al. 2016	MCC 2722; KCTC 39687; DSM 100104; VDS11	*Corynebacterium pollutisoli*	2535040	68.5	2430	Gp0156991	PRJEB20263	SAMN06295981	GCA_900177745	
Corynebacterium yudongzhengii 2183	Zhu et al. 2020	DSM 106264; CGMCC 1.16416	*Corynebacterium yudongzhengii*	2511302	64.9	2142		PRJNA431327	SAMN08388720	GCA_003065405	
Corynebacterium provencense SN15	Lo et al. 2019	DSM 101074; CSURP2161; Marseille-P2161	*Corynebacterium provencense*	3075769	66.9	2799		PRJEB12691	SAMEA3869306	GCA_900049755	
Corynebacterium bouchesdurhonense SN14	Lo et al. 2019	DSM 100846; CSURP2067; Marseille-P2067	*Corynebacterium bouchesdurhonense*	2255535	68.0	2147		PRJEB13138	SAMEA3905754	GCA_900078305	
Corynebacterium pseudotuberculosis ATCC 19410	(Buchanan 1911) Eberson 1918 emend. Nouioui et al. 2018	CCUG 2806; DSM 20689; JCM 9389; ATCC 19410; IFO 15363; NBRC 15363; NCTC 3450; CIP 102968	*Bacillus pseudotuberculosis*; *Corynebacterium pseudotuberculosis*	2337763	52.2	2146	Gp0223239	PRJNA382169	SAMN06701041	GCA_002155265	

Strain	Authority	Other deposits	Synonyms	Base pairs	Percent G+C	No. proteins	Goldstamp	Bioproject accession	Biosample accession	Assembly accession	IMG OID
Dermabacter jinjuensis 32	Park et al. 2016	DSM 101003; NCCP 16133	*Dermabacter jinjuensis*	2398 786	62.9	1996	Gp0266532	PRJNA407870	SAMN07665299	GCA_002443115	
Corynebacterium jeddahense JCB	Edouard et al. 2017	DSM 45997; CSUR P778	*Corynebacterium jeddahense*	2472 125	67.2	2341	Gp0101187	PRJEB4941	SAMEA3138931	GCA_000577555	
Corynebacterium pseudotuberculosis DSM 20689	(Buchanan 1911) Eberson 1918 emend. Nouioui et al. 2018	CCUG 2806; DSM 20689; JCM 9389; ATCC 19410; IFO 15363; NBRC 15363; NCTC 3450; CIP 102968	*Bacillus pseudotuberculosis*; *Corynebacterium pseudotuberculosis*	2338 546	52.2	2084	Gp0220522	PRJNA442833	SAMN08778220	GCA_003634885	2756170169
Corynebacterium ulcerans NCTC 7910	(ex Gilbert and Stewart 1927) Riegel et al. 1995	CCUG 2708; DSM 46325; JCM 10387; ATCC 51799; CIP 106504	*Corynebacterium ulcerans*	2453 761	53.3	2178	Gp0262745	PRJEB6403	SAMEA4504038	GCA_900187135	
Corynebacterium aquatimens DSM 45632	Aravena-Román et al. 2012	CCUG 61574; IMMIB L-2475	*Corynebacterium aquatimens*	2525 265	61.0	2230	Gp0305023				2880529280
Corynebacterium coyleae DSM 44184	Funke et al. 1997	CCUG 38194; JCM 10381; ATCC 700219; CIP 104919; DMMZ 214	*Corynebacterium coyleae*	2568 936	61.3	2419	Gp0116505	PRJNA303722	SAMN04488531	GCA_900105505	2634166170

Strain	Authority	Other deposits	Synonyms	Base pairs	Percent G+C	No. proteins	Goldstamp	Bioproject accession	Biosample accession	Assembly accession	IMG OID
Corynebacterium mycetoides DSM 20632	(ex Castellani 1942) Collins 1983 emend. Nouioui et al. 2018	CCUG 27538; JCM 9388; ATCC 43995; IFO 15289; NBRC 15289; NCTC 9864; CIP 55.51	*Corynebacterium mycetoides*	2266 370	66.6	2129	Gp0116506	PRJNA303721	SAMN04488535	GCA_900103625	2634166344
Corynebacterium accolens ATCC 49725	Neubauer et al. 1991 emend. Nouioui et al. 2018	CNCTC Th 1/57; CCUG 28779; DSM 44278; JCM 8331; CIP 104783	*Corynebacterium accolens*	2406 629	59.7	2333	Gp0003417	PRJNA31443	SAMN00002226	GCA_000159115	643886058
Corynebacterium striatum ATCC 6940	(Chester 1901) Eberson 1918 emend. Nouioui et al. 2018	CCUG 27949; DSM 20668; JCM 9390; ATCC 6940; IFO 15291; NBRC 15291; NCTC 764; CIP 81.15	*Bacterium striatum*; *Corynebacterium striatum*	2717 381	59.4	2677	Gp0003467	PRJNA31449	SAMN00001507	GCA_000159135	643886057
Corynebacterium amycolatum ATCC 49368	Collins et al. 1988	CCUG 35685; DSM 6922; JCM 7447; IFO 15207; NBRC 15207; CIP 103452; NCFB 2768; NCIMB 13130; S160	*Corynebacterium amycolatum*	2448 224	58.7	2128		PRJNA224116	SAMN07741515	GCA_014335175	
Corynebacterium auriscanis CIP 106629	Collins et al. 2000 emend. Nouioui et al. 2018	CCUG 39938; DSM 44609; JCM 12369; M598/96/1	*Corynebacterium auriscanis*	2568 862	58.5	1537	Gp0107708	PRJNA262562	SAMN03106126	GCA_000767255	

Strain	Authority	Other deposits	Synonyms	Base pairs	Percent G+C	No. proteins	Goldstamp	Bioproject accession	Biosample accession	Assembly accession	IMG OID
Corynebacterium humireducens DSM 45392	Wu et al. 2011 emend. Nouioui et al. 2018	DSM 45392; NBRC 106098; CGMCC 2452; MFC-5	*Corynebacterium humireducens*	2681 312	68.6	2545	Gp0023681	PRJNA172965	SAMN03283197	GCA_000819445	
Corynebacterium imitans DSM 44264	Funke et al. 1997 emend. Nouioui et al. 2018	2023; CCUG 36877; DSM 44264; JCM 10386; ATCC 700354; IFO 16163; NBRC 16163; NBRC 100416; NCTC 13015; CIP 105130	*Corynebacterium imitans*	2565 109	64.3	2013	Gp0094474	PRJNA246650	SAMN02950575	GCA_000739455	
Corynebacterium kutscheri DSM 20755	(Migula 1900) Bergey et al. 1925 emend. Nouioui et al. 2018	CCUG 27535; DSM 20755; JCM 9385; ATCC 15677; IFO 15288; NBRC 15288; NCTC 11138; CIP 103423	*Bacterium kutscheri*; *Corynebacterium kutscheri*	2354 065	46.5	2047	Gp0110293	PRJNA276037	SAMN03365283	GCA_000980835	
Corynebacterium marinum DSM 44953	Du et al. 2010 emend. Nouioui et al. 2018	7015; NRRL B-24779; DSM 44953; CGMCC 1.6998; D7015	*Corynebacterium marinum*	2729 219	67.8	2550	Gp0023707	PRJNA172963	SAMN02800399	GCA_000835165	
Corynebacterium mustelae DSM 45274	Funke et al. 2010 emend. Nouioui et al. 2018	3105; CCUG 57279	*Corynebacterium mustelae*	3474 226	52.6	3110	Gp0114696	PRJNA282348	SAMN03568800	GCA_001020985	

Strain	Authority	Other deposits	Synonyms	Base pairs	Percent G+C	No. proteins	Goldstamp	Bioproject accession	Biosample accession	Assembly accession	IMG OID
Corynebacterium singulare DSM 44357	Riegel et al. 1997 emend. Nouioui et al. 2018	CCUG 37330; JCM 10385; IFO 16162; NBRC 16162; CIP 105491; IBS B52218	*Corynebacterium singulare*	2830 499	60.1	2561	Gp0109683	PRJNA246651	SAMN03177398	GCA_000833575	
Corynebacterium testudinoris DSM 44614	Collins et al. 2001 emend. Nouioui et al. 2018	CCUG 41823; JCM 12108; CIP 106763; M935/96/4	*Corynebacterium testudinoris*	2721 226	63.1	2560	Gp0114695	PRJNA280910	SAMN03480629	GCA_001021045	
Corynebacterium ureicelerivorans DSM 45051	Yassin 2007 emend. Nouioui et al. 2018	CCUG 53377; JCM 15295; IMMIB RIV-2301	*Corynebacterium ureicelerivorans*	2328 188	65.0	1922	Gp0103378	PRJNA257688	SAMN02953970	GCA_000747315	
Corynebacterium uterequi DSM 45634	Hoyles et al. 2013 emend. Nouioui et al. 2018	CCUG 61235; VM 2298	*Corynebacterium uterequi*	2419 437	65.5	2163	Gp0114697	PRJNA280912	SAMN03480647	GCA_001021065	
Corynebacterium lactis DSM 45799	Wiertz et al. 2013 emend. Nouioui et al. 2018	CCUG 63372; RW2-5	*Corynebacterium lactis*	2769 745	60.5	2364	Gp0067969	PRJNA222474	SAMN04012704	GCA_001274895	
Corynebacterium glyciniphilum ATCC 21341	(ex Kubota et al. 1972) Al-Dilaimi et al. 2015	AJ 3170; DSM 45795	*Corynebacterium glyciniphilum*	3568 218	64.8	3270	Gp0047724	PRJNA221205	SAMN03081498	GCA_000626675	2576861442
Corynebacterium epidermidicanis DSM 45586	Frischmann et al. 2012 emend. Nouioui et al. 2018	410; LMG 26322; CCUG 60915	*Corynebacterium epidermidicanis*	2692 072	58.1	2465	Gp0114694	PRJNA280479	SAMN03462986	GCA_001021025	
Corynebacterium minutissimum ATCC 23348	(ex Sarkany et al. 1962) Collins and Jones 1983 emend. Nouioui et al. 2018	CCUG 541; DSM 20651; JCM 9387; ATCC 23348; IFO 15361; NBRC 15361; NCTC 10288; CIP 100652	*Corynebacterium minutissimum*	2663 401	60.0	2276	Gp0122002	PRJNA264738	SAMN03140311	GCA_000805675	

Strain	Authority	Other deposits	Synonyms	Base pairs	Percent G+C	No. proteins	Goldstamp	Bioproject accession	Biosample accession	Assembly accession	IMG OID
Corynebacterium minutissimum NBRC 15361	(ex Sarkany et al. 1962) Collins and Jones 1983 emend. Nouioui et al. 2018	CCUG 541; DSM 20651; JCM 9387; ATCC 23348; IFO 15361; NBRC 15361; NCTC 10288; CIP 100652	*Corynebacterium minutissimum*	2663 455	60.0	2464	Gp0024448	PRJDB438	SAMD00046517	GCA_001552395	
Corynebacterium halotolerans YIM 70093	Chen et al. 2004 emend. Nouioui et al. 2018	DSM 44683; JCM 12676; CCTCC AA 001024; YIM 70093	*Corynebacterium halotolerans*	3222 002	68.3	2865	Gp0023456	PRJNA168616	SAMN02603027	GCA_000341345	2524023198
Corynebacterium xerosis NBRC 16721	(Lehmann and Neumann 1896) Lehmann and Neumann 1899 emend. Nouioui et al. 2018	CCUG 27544; DSM 20743; JCM 1971; ATCC 373; IFO 16721; NBRC 16721; NCTC 11861; CIP 100653	*Bacillus xerosis*; *Corynebacterium xerosis*	2686 219	69.7	2351	Gp0024449	PRJDB439	SAMD00046521	GCA_001552415	
Corynebacterium nuruki S6-4	Shin et al. 2011 emend. Nouioui et al. 2018	DSM 45695; JCM 17162; KACC 15032	*Corynebacterium nuruki*	3106 595	69.5	2787	Gp0011122	PRJNA66913	SAMN02470217	GCA_000213935	2547132106
Corynebacterium glutamicum ATCC 13032	(Kinoshita et al. 1958) Abe et al. 1967 emend. Nouioui et al. 2018	LMG 3730; NRRL B-2784; CCUG 27702; DSM 20300; JCM 1318; IFO 12168; NBRC 12168; CIP 82.08; HAMBI 2052	*Corynebacterium glutamicum*; *Micrococcus glutamicus*	3282 708	53.8	3057	Gp0000615	PRJNA13760	SAMEA3138338	GCA_000196335	639279306

Strain	Authority	Other deposits	Synonyms	Base pairs	Percent G+C	No. proteins	Goldstamp	Bioproject accession	Biosample accession	Assembly accession	IMG OID
Corynebacterium urealyticum DSM 7109	Pitcher et al. 1992 emend. Nouioui et al. 2018	LMG 19041; CCUG 18158; DSM 7109; JCM 10395; ATCC 43042; NCTC 12011; CIP 103524	*Corynebacterium urealyticum*	2369 219	64.2	2024	Gp0001357	PRJNA29211	SAMEA3138282	GCA_000069945	641522620
Corynebacterium marinum CGMCC 1.6998	Du et al. 2010 emend. Nouioui et al. 2018	7015; NRRL B-24779; DSM 44953; CGMCC 1.6998; D7015	*Corynebacterium marinum*	2680 020	67.9	2569		PRJDB10509	SAMD00245145	GCA_014645275	
Dermabacter vaginalis AD1-86	Chang et al. 2016	KCTC 39585; DSM 100050	*Dermabacter vaginalis*	2392 314	62.6	2129	Gp0203766	PRJNA286956	SAMN03774729	GCA_001678905	
Rhodococcus opacus DSM 43205	Klatte et al. 1995	DSM 43205; JCM 9703; ATCC 51881; IFO 16217; NBRC 16217; NBRC 100624; CIP 104549	*Rhodococcus opacus*	8534 314	67.3	7425	Gp0150346	PRJNA224116	SAMN04357312	GCF_001646735	
Corynebacterium pacaense Marseille-P2417 T	Bellali et al. 2019	CSUR P2417	*Corynebacterium pacaense*	3027 822	63.7	2736		PRJEB19973	SAMEA103910525	GCA_900169525	

Strain	Authority	Other deposits	Synonyms	Base pairs	Percent G+C	No. proteins	Goldstamp	Bioproject accession	Biosample accession	Assembly accession	IMG OID
Corynebacterium pilosum CIP 103422	Yanagawa and Honda 1978	CCUG 27193; DSM 20521; JCM 3714; ATCC 29592; IFO 15285; NBRC 15285; NCTC 11862; CIP 103422	*Corynebacterium pilosum*	2545 970	60.7	2772	Gp0120674	PRJNA284680	SAMN03731012	GCA_001044155	
Corynebacterium bovis DSM 20582	Bergey et al. 1923	CCUG 2705; JCM 11947; ATCC 7715; NCTC 3224; CIP 54.80	*Corynebacterium bovis*	2694 851	72.9	2169	Gp0325157	PRJNA546935	SAMN12024754	GCA_014191555	2824244109
Corynebacterium dentalis Marseille-P4122	Benabdelkader et al. 2020	CSURP412 2	*Corynebacterium dentalis*	2302 937	59.9	2080		PRJNA224116	SAMEA104348950	GCF_900232865	
Corynebacterium lowii LMG 28276	Bernard et al. 2016	CCUG 65815; NML 130206; R-50085; TKD4	*Corynebacterium lowii*	2354 433	62.9	2108		PRJNA224116	SAMN04091594	GCF_001412085	
Corynebacterium durum DSM 45333	Riegel et al. 1997	CCUG 37331; DSM 44351; JCM 11948; CIP 105490; IBS G15036	*Corynebacterium durum*	2800 016	57.2	2613	Gp0456002				2856490523
Corynebacterium liangguodongii 2184	Zhu et al. 2020	DSM 106203; CGMCC 1.16417	*Corynebacterium liangguodongii*	2357 924	66.1	2115		PRJNA431327	SAMN08388739	GCA_003070865	

Strain	Authority	Other deposits	Synonyms	Base pairs	Percent G+C	No. proteins	Goldstamp	Bioproject accession	Biosample accession	Assembly accession	IMG OID
Corynebacterium jeikeium ATCC 43734	Jackman et al. 1988 emend. Nouioui et al. 2018	CCUG 27192; DSM 46361; DSM 7171; JCM 9384; ATCC 43734; NCTC 11913; CIP 103337	*Corynebacterium jeikeium*	2425 907	61.6	2224	Gp0004510	PRJNA31445	SAMN00001506	GCA_000163435	647000231
Corynebacterium neomassiliense Marseille-P3888	Boxberger et al. 2020	CCUG7235 2; CSURP388 8	*Corynebacterium neomassiliense*	3139 653	66.9	2729		PRJNA224116	SAMEA5140071	GCF_900626215	
Corynebacterium diphtheriae NCTC 11397	(Kruse 1886) Lehmann and Neumann 1896 emend. Nouioui et al. 2018	DSM 44123; ATCC 27010; NCTC 11397; CIP 100721	*Bacillus diphtheriae*; *Corynebacterium diphtheriae*; *Corynebacterium diphtheriae* subsp. *diphtheriae*	2463 666	53.5	2337	Gp0132011	PRJEB6403	SAMEA2517360	GCA_001457455	
Corynebacterium argentoratense DSM 44202	Riegel et al. 1995 emend. Nouioui et al. 2018	CCUG 34893; JCM 10392; ATCC 51927; CIP 104296; IBS B10697	*Corynebacterium argentoratense*	2031 862	58.9	1875	Gp0044215	PRJNA209048	SAMN02603032	GCA_000590555	2554235426
Corynebacterium terpenotabidum Y-11	Takeuchi et al. 1999	DSM 44721; JCM 10555; IFO 14764; NBRC 14764; VKM Ac-2071; CIP 105927	*Corynebacterium terpenotabidum*	2751 233	67.0	2369	Gp0022753	PRJNA168617	SAMN02603028	GCA_000418365	2554235357
Ckutscheri_DSM 20755.fna				2354 065	46.5	2113					
Clactis_RW25.fn a				2769 745	60.5	2455					
Cmarinum_DSM 44953.fna				2729 219	67.8	2543					

Strain	Authority	Other deposits	Synonyms	Base pairs	Percent G+C	No. proteins	Goldstamp	Bioproject accession	Biosample accession	Assembly accession	IMG OID
Cmaris_DSM45190.fna				2833547	66.6	2608					
Cmustelae_DSM45274.fna				3474226	52.6	3146					
Cpseudotuberculosis_31.fna				2297010	52.2	2123					
Cpseudotuberculosis_ATCC19410.fna				2337763	52.2	2144					
CriegeliiPUDD83A45.fna				2563723	60.5	2402					
Crouxii_FRC0190.fna				2451019	53.2	2366					
Csilvaticum_KL0182.fna				2553200	54.4	2537					
Csimulans_PES1.fna				2737971	59.0	2566					
Csingulare_IBSB52218.fna				2830519	60.1	2596					
Cterpenotabidum_Y11.fna				2751233	67.0	2406					
Ctestudinoris_DSM44614.fna				2721226	63.1	2577					
Culcerans_NCTC7910.fna				2453761	53.3	2207					
Culcerans_NCTC12077.fna				2616289	53.4	2454					
Curealyticum_DSM7109.fna				2369219	64.2	2011					
Cureicelerivorans_IMMIBRIV2301.fna				2328278	65.0	2298					
Cuterequi_DSM45634.fna				2419437	65.5	2184					
Cvitaeruminis_DSM20294.fna				2931780	65.5	2604					

Methods, Results and References

The genome sequence data were uploaded to the Type (Strain) Genome Server (TYGS), a free bioinformatics platform available under https://tygs.dsmz.de, for a whole genome-based taxonomic analysis [1]. The results were provided by the TYGS on 2021-02-09. The TYGS analysis was subdivided into the following steps:

Determination of closely related type strains

Determination of closest type strain genomes was done in two complementary ways: First, all user genomes were compared against all type strain genomes available in the TYGS database via the MASH algorithm, a fast approximation of intergenomic relatedness [2], and, the ten type strains with the smallest MASH distances chosen per user genome. Second, an additional set of ten closely related type strains was determined via the 16S rDNA gene sequences. These were extracted from the user genomes using RNAmmer [3] and each sequence was subsequently BLASTed [4] against the 16S rDNA gene sequence of each of the currently 14130 type strains available in the TYGS database. This was used as a proxy to find the best 50 matching type strains (according to the bitscore) for each user genome and to subsequently calculate precise distances using the Genome BLAST Distance Phylogeny approach (GBDP) under the algorithm 'coverage' and distance formula d_5 [5]. These distances were finally used to determine the 10 closest type strain genomes for each of the user genomes.

Pairwise comparison of genome sequences

For the phylogenomic inference, all pairwise comparisons among the set of genomes were conducted using GBDP and accurate intergenomic distances inferred under the algorithm 'trimming' and distance formula d_5 [5]. 100 distance replicates were calculated each. Digital DDH values and confidence intervals were calculated using the recommended settings of the GGDC 2.1 [5].

Phylogenetic inference

The resulting intergenomic distances were used to infer a balanced minimum evolution tree with branch support via FASTME 2.1.4 including SPR postprocessing [6]. Branch support was inferred from 100 pseudo-bootstrap replicates each. The trees were rooted at the midpoint [7] and visualized with PhyD3 [8].

Type-based species and subspecies clustering

The type-based species clustering using a 70% dDDH radius around each of the 103 type strains was done as previously described [1]. The resulting groups are shown in Table 1 and 4. Subspecies clustering was done using a 79% dDDH threshold as previously introduced [9].

Results

Type-based species and subspecies clustering

The resulting species and subspecies clusters are listed in Table 4, whereas the taxonomic identification of the query strains is found in Table 1. Briefly, the clustering yielded 85 species clusters and the provided query strains were assigned to 19 of these. Moreover, user strains were located in 19 of 87 subspecies clusters.

Figure caption SSU tree

Figure 1. Tree inferred with FastME 2.1.6.1 [6] from GBDP distances calculated from 16S rDNA gene sequences. The branch lengths are scaled in terms of GBDP distance formula d_5. The numbers above branches are GBDP pseudo-bootstrap support values > 60 % from 100 replications, with an average branch support of 76.6 %. The tree was rooted at the midpoint [7].

Figure caption genome tree

Figure 2. Tree inferred with FastME 2.1.6.1 [6] from GBDP distances calculated from genome sequences. The branch lengths are scaled in terms of GBDP distance formula d_5. The numbers above branches are GBDP pseudo-bootstrap support values > 60 % from 100 replications, with an average branch support of 41.3 %. The tree was rooted at the midpoint [7].

References

[1] Meier-Kolthoff JP, Göker M. TYGS is an automated high-throughput platform for state-of-the-art genome-based taxonomy. Nat. Commun. 2019;10: 2182. DOI: 10.1038/s41467-019-10210-3

[2] Ondov BD, Treangen TJ, Melsted P, et al. Mash: Fast genome and metagenome distance estimation using MinHash. Genome Biol 2016;17: 1–14. DOI: 10.1186/s13059-016-0997-x

[3] Lagesen K, Hallin P. RNAmmer: consistent and rapid annotation of ribosomal RNA genes. Nucleic Acids Res. Oxford Univ Press; 2007;35: 3100–3108. DOI: 10.1093/nar/gkm160

[4] Camacho C, Coulouris G, Avagyan V, Ma N, Papadopoulos J, Bealer K, et al. BLAST+: architecture and applications. BMC Bioinformatics. 2009;10: 421. DOI: 10.1186/1471-2105-10-421

[5] Meier-Kolthoff JP, Auch AF, Klenk H-P, Göker M. Genome sequence-based species delimitation with confidence intervals and improved distance functions. BMC Bioinformatics. 2013;14: 60. DOI: 10.1186/1471-2105-14-60

[6] Lefort V, Desper R, Gascuel O. FastME 2.0: A comprehensive, accurate, and fast distance-based phylogeny inference program. Mol Biol Evol. 2015;32: 2798–2800. DOI: 10.1093/molbev/msv150

[7] Farris JS. Estimating phylogenetic trees from distance matrices. Am Nat. 1972;106: 645–667.

[8] Kreft L, Botzki A, Coppens F, Vandepoele K, Van Bel M. PhyD3: A phylogenetic tree viewer with extended phyloXML support for functional genomics data visualization. Bioinformatics. 2017;33: 2946–2947. DOI: 10.1093/bioinformatics/btx324

[9] Meier-Kolthoff JP, Hahnke RL, Petersen J, Scheuner C, Michael V, Fiebig A, et al. Complete genome sequence of DSM 30083^{T}, the type strain (U5/41^{T}) of *Escherichia coli*, and a proposal for delineating subspecies in microbial taxonomy. Stand Genomic Sci. 2014;9: 2. DOI: 10.1186/1944-3277-9-2

TYPE (STRAIN) GENOME SERVER

JOB SUMMARY

PRINT DATE: 2021-02-09 14:52:32 +0100

JOB ID: f569001b-816c-42f6-bbef-97f15346caa3

RESULT PAGE: https://tygs.dsmz.de/user_results/show?guid=f569001b-816c-42f6-bbef-97f15346caa3

Table 1: Phylogenies

Publication-ready versions of both the genome-scale GBDP tree and the 16S rRNA gene sequence tree can be customized and exported either in SVG (vector graphic) or PNG format from within the phylogeny viewers in your TYGS result page. For publications the **SVG format is recommended** because it is lossless, always keeps its high resolution and can also be easily converted to other popular formats such as PDF or EPS. Please follow the link provided above!

Table 2: Identification

The below list contains the result of the TYGS species identification routine.

Explanation of remarks that might occur in the below table:

remark [R1]: The TYGS type strain database is automatically updated on an almost daily basis. However, if a particular type strain genome is not available in the TYGS database, this can have several reasons which are detailed in the FAQ. You can request an extended 16S rRNA gene analysis via the 16S tree viewer found in your result page to detect **not yet genome-sequenced** type strains relevant for your study.

remark [R2]: > 70% dDDH value (formula d_4) and (almost) minimal dDDH values for gene-content formulae d_0 and d_6 indicate a potentially unreliable identification result and should thus be checked via the 16S rRNA gene sequence similarity. Such strong deviations can, in principle, be caused by sequence contamination.

remark [R3]: G+C content difference of > 1 % indicates a potentially unreliable identification result because within species G+C content varies no more than 1 %, if computed from genome sequences (PMID: 24505073).

Strain	Conclusion	Identification result	Remark
**'Mtuberculosis_H37Rv'**	belongs to known species	*Mycobacterium tuberculosis*	

Table 3: Pairwise comparisons of user genomes vs. type-strain genomes

The following table contains the pairwise dDDH values between your user genomes and the selected type-strain genomes. The dDDH values are provided along with their confidence intervals (C.I.) for the three different GBDP formulas:

- formula d_0 (a.k.a. GGDC formula 1): length of all HSPs divided by total genome length
- formula d_4 (a.k.a. GGDC formula 2): sum of all identities found in HSPs divided by overall HSP length
- formula d_6 (a.k.a. GGDC formula 3): sum of all identities found in HSPs divided by total genome length

Note: Formula d_4 is independent of genome length and is thus robust against the use of incomplete draft genomes. For other reasons for preferring formula d_4, see the FAQ.

Query	Subject	d_0	C.I. d_0	d_4	C.I. d_4	d_6	C.I. d_6	Diff. G+C Percent
**'Mtuberculosis_H37Rv.fna'**	*Mycobacterium tuberculosis* H37Rv	100.0	[100.0 - 100.0]	100.0	[100.0 - 100.0]	100.0	[100.0 - 100.0]	0.0
**'Mtuberculosis_H37Rv.fna'**	*Mycobacterium microti* ATCC 19422	98.6	[97.6 - 99.2]	98.7	[98.0 - 99.1]	99.3	[98.8 - 99.6]	0.27
**'Mtuberculosis_H37Rv.fna'**	*Mycobacterium caprae* ATCC BAA-824	99.5	[99.0 - 99.8]	97.9	[97.0 - 98.6]	99.7	[99.4 - 99.8]	0.04
**'Mtuberculosis_H37Rv.fna'**	*Mycobacterium africanum* ATCC 25420	99.6	[99.3 - 99.8]	97.8	[96.9 - 98.5]	99.8	[99.6 - 99.9]	0.07
**'Mtuberculosis_H37Rv.fna'**	*Mycobacterium bovis* ATCC 19210	99.4	[98.8 - 99.7]	97.5	[96.5 - 98.2]	99.6	[99.3 - 99.8]	0.06
**'Mtuberculosis_H37Rv.fna'**	*Mycobacterium pinnipedii* ATCC BAA-688	99.7	[99.4 - 99.9]	97.4	[96.3 - 98.1]	99.8	[99.6 - 99.9]	0.02
**'Mtuberculosis_H37Rv.fna'**	*Mycobacterium decipiens* TBL 1200985	45.8	[42.5 - 49.3]	30.2	[27.8 - 32.7]	41.2	[38.2 - 44.2]	0.1
**'Mtuberculosis_H37Rv.fna'**	*Mycobacterium shinjukuense* CCUG 53584	39.3	[36.0 - 42.8]	25.8	[23.5 - 28.3]	34.9	[31.9 - 38.0]	2.14
**'Mtuberculosis_H37Rv.fna'**	*Mycobacterium shinjukuense* JCM 14233	40.0	[36.6 - 43.4]	25.7	[23.4 - 28.2]	35.3	[32.4 - 38.4]	2.17
**'Mtuberculosis_H37Rv.fna'**	*Mycobacterium lacus* JCM 15657	33.6	[30.2 - 37.2]	24.8	[22.5 - 27.3]	30.4	[27.5 - 33.5]	1.33
**'Mtuberculosis_H37Rv.fna'**	*Mycobacterium marinum* DSM 44344	21.1	[17.9 - 24.7]	22.2	[19.9 - 24.6]	20.4	[17.6 - 23.4]	0.08
**'Mtuberculosis_H37Rv.fna'**	*Mycobacterium marinum* NCTC 2275	21.0	[17.8 - 24.6]	22.1	[19.9 - 24.6]	20.2	[17.5 - 23.3]	0.05
**'Mtuberculosis_H37Rv.fna'**	*Mycobacterium marinum* CCUG 20998	21.1	[17.9 - 24.8]	22.1	[19.8 - 24.6]	20.4	[17.6 - 23.4]	0.1

Table 4: Strains in your dataset

Joint dataset of automatically determined closest type strains (if this mode was chosen), manually selected type strains (if selected accordingly) and the provided user strains, if provided (marked in yellow).

Strain	Authority	Other deposits	Synonyms	Base pairs	Percent G+C	No. proteins	Goldstamp	Bioproject accession	Biosample accession	Assembly accession	IMG OID
Mycobacterium bovis ATCC 19210	Karlson and Lessel 1970	NCTC 10772; CIP 105234	*Mycobacterium bovis*; *Mycobacterium bovis* subsp. *bovis*	4303 498	65.6	4048		PRJNA377261	SAMN06462278	GCA_002982285	
Mycobacterium africanum ATCC 25420	Castets et al. 1969	CIP 105147	*Mycobacterium africanum*	4351 982	65.5	4043		PRJNA224116	SAMN06462277	GCF_002982335	
Mycobacterium pinnipedii ATCC BAA-688	Cousins et al. 2003	6482; NCTC 13288	*Mycobacterium pinnipedii*	4324 277	65.6	4036		PRJNA224116	SAMN06462281	GCF_002982275	
Mycobacterium marinum CCUG 20998	Aronson 1926	CCUG 20998; CCUG 27843; DSM 43225; DSM 44344; JCM 12275; ATCC 927; NCTC 2275; CIP 104528	*Mycobacterium marinum*	6453 310	65.7	5434	Gp0371789	PRJNA414525	SAMN07792364	GCA_003391395	
Mycobacterium tuberculosis H37Rv	(Zopf 1883) Lehmann and Neumann 1896 emend. Riojas et al. 2018	ATCC 27294	*Bacterium tuberculosis*; *Mycobacterium tuberculosis*; *Mycobacterium tuberculosis* subsp. *tuberculosis*	4411 532	65.6	4018	Gp0000774	PRJNA224	SAMEA3138326	GCA_000195955	637000173
Mycobacterium shinjukuense JCM 14233	Saito et al. 2011 emend. Nouioui et al. 2018	CCUG 53584; DSM 45663; JCM 14233; GTC 2738	*Mycobacterium shinjukuense*	4504 020	67.8	4119		PRJDB7717	SAMD00153190	GCA_010730055	

Strain	Authority	Other deposits	Synonyms	Base pairs	Percent G+C	No. proteins	Goldstamp	Bioproject accession	Biosample accession	Assembly accession	IMG OID
Mycobacterium lacus JCM 15657	Turenne et al. 2002 emend. Nouioui et al. 2018	DSM 44577; JCM 15657; ATCC BAA-323; NRCM 00-255	*Mycobacterium lacus*	5092 988	66.9	4794		PRJDB7717	SAMD00153198	GCA_010731535	
Mycobacterium marinum DSM 44344	Aronson 1926	CCUG 20998; CCUG 27843; DSM 43225; DSM 44344; JCM 12275; ATCC 927; NCTC 2275; CIP 104528	*Mycobacterium marinum*	6269 462	65.7	5516		PRJNA414948	SAMN07811439	GCA_003431645	
Mycobacterium marinum NCTC 2275	Aronson 1926	CCUG 20998; CCUG 27843; DSM 43225; DSM 44344; JCM 12275; ATCC 927; NCTC 2275; CIP 104528	*Mycobacterium marinum*	6318 015	65.7	5601		PRJNA414948	SAMN07811438	GCA_003431655	
Mycobacterium caprae ATCC BAA-824	(Aranaz et al. 1999) Aranaz et al. 2003	CIP 105776; gM-1; spc-1	*Mycobacterium bovis* subsp. *caprae*; *Mycobacterium caprae*; *Mycobacterium tuberculosis* subsp. *caprae*	4304 249	65.6	4039		PRJNA224116	SAMN06462279	GCF_002982225	
Mycobacterium microti ATCC 19422	Reed 1957	DSM 44155; NCTC 8710; CIP 104256	*Mycobacterium microti*	4241 638	65.3	4102		PRJNA224116	SAMN06462280	GCF_002982215	

Strain	Authority	Other deposits	Synonyms	Base pairs	Percent G+C	No. proteins	Goldstamp	Bioproject accession	Biosample accession	Assembly accession	IMG OID
Mycobacterium shinjukuense CCUG 53584	Saito et al. 2011 emend. Nouioui et al. 2018	CCUG 53584; DSM 45663; JCM 14233; GTC 2738	*Mycobacterium shinjukuense*	4409896	67.8	3701		PRJNA224116	SAMN06064260	GCF_002086755	
Mycobacterium decipiens TBL 1200985	Brown-Elliott et al. 2018	DSM 105360; ATCC TSD-117	*Mycobacterium decipiens*	5216890	65.5	4492		PRJNA354248	SAMN06651657	GCA_002104675	
Mtuberculosis_H37Rv.fna				4411709	65.6	4079					

Methods, Results and References

The genome sequence data were uploaded to the Type (Strain) Genome Server (TYGS), a free bioinformatics platform available under https://tygs.dsmz.de, for a whole genome-based taxonomic analysis [1]. The results were provided by the TYGS on 2021-02-09. The TYGS analysis was subdivided into the following steps:

Determination of closely related type strains

Determination of closest type strain genomes was done in two complementary ways: First, all user genomes were compared against all type strain genomes available in the TYGS database via the MASH algorithm, a fast approximation of intergenomic relatedness [2], and, the ten type strains with the smallest MASH distances chosen per user genome. Second, an additional set of ten closely related type strains was determined via the 16S rDNA gene sequences. These were extracted from the user genomes using RNAmmer [3] and each sequence was subsequently BLASTed [4] against the 16S rDNA gene sequence of each of the currently 14130 type strains available in the TYGS database. This was used as a proxy to find the best 50 matching type strains (according to the bitscore) for each user genome and to subsequently calculate precise distances using the Genome BLAST Distance Phylogeny approach (GBDP) under the algorithm 'coverage' and distance formula d_5 [5]. These distances were finally used to determine the 10 closest type strain genomes for each of the user genomes.

Pairwise comparison of genome sequences

For the phylogenomic inference, all pairwise comparisons among the set of genomes were conducted using GBDP and accurate intergenomic distances inferred under the algorithm 'trimming' and distance formula d_5 [5]. 100 distance replicates were calculated each. Digital DDH values and confidence intervals were calculated using the recommended settings of the GGDC 2.1 [5].

Phylogenetic inference

The resulting intergenomic distances were used to infer a balanced minimum evolution tree with branch support via FASTME 2.1.4 including SPR postprocessing [6]. Branch support was inferred from 100 pseudo-bootstrap replicates each. The trees were rooted at the midpoint [7] and visualized with PhyD3 [8].

Type-based species and subspecies clustering

The type-based species clustering using a 70% dDDH radius around each of the 13 type strains was done as previously described [1]. The resulting groups are shown in Table 1 and 4. Subspecies clustering was done using a 79% dDDH threshold as previously introduced [9].

Results

Type-based species and subspecies clustering

The resulting species and subspecies clusters are listed in Table 4, whereas the taxonomic identification of the query strains is found in Table 1. Briefly, the clustering yielded 5 species clusters and the provided query strains were assigned to 1 of these. Moreover, user strains were located in 1 of 5 subspecies clusters.

Figure caption SSU tree

Figure 1. Tree inferred with FastME 2.1.6.1 [6] from GBDP distances calculated from 16S rDNA gene sequences. The branch lengths are scaled in terms of GBDP distance formula d_5. The numbers above branches are GBDP pseudo-bootstrap support values > 60 % from 100 replications, with an average branch support of 43.0 %. The tree was rooted at the midpoint [7].

Figure caption genome tree

Figure 2. Tree inferred with FastME 2.1.6.1 [6] from GBDP distances calculated from genome sequences. The branch lengths are scaled in terms of GBDP distance formula d_5. The numbers above branches are GBDP pseudo-bootstrap support values > 60 % from 100 replications, with an average branch support of 48.9 %. The tree was rooted at the midpoint [7].

References

[1] Meier-Kolthoff JP, Göker M. TYGS is an automated high-throughput platform for state-of-the-art genome-based taxonomy. Nat. Commun. 2019;10: 2182. DOI: 10.1038/s41467-019-10210-3

[2] Ondov BD, Treangen TJ, Melsted P, et al. Mash: Fast genome and metagenome distance estimation using MinHash. Genome Biol 2016;17: 1–14. DOI: 10.1186/s13059-016-0997-x

[3] Lagesen K, Hallin P. RNAmmer: consistent and rapid annotation of ribosomal RNA genes. Nucleic Acids Res. Oxford Univ Press; 2007;35: 3100–3108. DOI: 10.1093/nar/gkm160

[4] Camacho C, Coulouris G, Avagyan V, Ma N, Papadopoulos J, Bealer K, et al. BLAST+: architecture and applications. BMC Bioinformatics. 2009;10: 421. DOI: 10.1186/1471-2105-10-421

[5] Meier-Kolthoff JP, Auch AF, Klenk H-P, Göker M. Genome sequence-based species delimitation with confidence intervals and improved distance functions. BMC Bioinformatics. 2013;14: 60. DOI: 10.1186/1471-2105-14-60

[6] Lefort V, Desper R, Gascuel O. FastME 2.0: A comprehensive, accurate, and fast distance-based phylogeny inference program. Mol Biol Evol. 2015;32: 2798–2800. DOI: 10.1093/molbev/msv150

[7] Farris JS. Estimating phylogenetic trees from distance matrices. Am Nat. 1972;106: 645–667.

[8] Kreft L, Botzki A, Coppens F, Vandepoele K, Van Bel M. PhyD3: A phylogenetic tree viewer with extended phyloXML support for functional genomics data visualization. Bioinformatics. 2017;33: 2946–2947. DOI: 10.1093/bioinformatics/btx324

[9] Meier-Kolthoff JP, Hahnke RL, Petersen J, Scheuner C, Michael V, Fiebig A, et al. Complete genome sequence of DSM 30083^T, the type strain (U5/41^T) of *Escherichia coli*, and a proposal for delineating subspecies in microbial taxonomy. Stand Genomic Sci. 2014;9: 2. DOI: 10.1186/1944-3277-9-2
